# Feasibility and acute physiological responses to supramaximal high-intensity interval training in COPD: a randomised crossover trial

**DOI:** 10.1183/23120541.01321-2024

**Published:** 2025-09-22

**Authors:** Johan Jakobsson, Jana De Brandt, Mattias Hedlund, Anna-Clara Rullander, Thomas Sandström, André Nyberg

**Affiliations:** 1 Department of Community Medicine and Rehabilitation, Umeå University, Umeå, Sweden; 2 Department of Nursing, Umeå University, Umeå, Sweden; 3 Department of Public Health and Clinical Medicine, Umeå University, Umeå, Sweden; 4 These authors contributed equally

## Abstract

**Background:**

Extrapulmonary manifestations, including cognitive impairment and reduced muscle and cardiovascular function is common in COPD. While high-intensity exercise offers extrapulmonary benefits, its implementation in COPD is challenging. This randomised crossover trial examined the feasibility and physiological responses of a novel supramaximal high-intensity interval-training (SupraHIIT) protocol compared with moderate-intensity continuous training (MICT) in people with COPD and matched healthy controls (HCs).

**Methods:**

16 people with COPD and 16 HCs performed SupraHIIT and MICT. SupraHIIT consisted of ten 6-s intervals at ≈150% and ≈200% of maximum aerobic power (MAP), while MICT was performed for 20 min at 60% of MAP. Outcomes were exercise intensity, change in exerkines, feasibility and cardiorespiratory demand of the modalities.

**Results:**

SupraHIIT was feasible and enabled up to a 3.5-fold increase in external exercise intensity compared with MICT (184±66 and 245±88 *versus* 71±22 W in COPD; p<0.001). All participants could complete SupraHIIT, which was the preferred modality in both groups (p<0.01), whereas 5 of 16 participants with COPD interrupted MICT due to intolerable dyspnoea or exhaustion (p=0.005). Both modalities increased plasma brain-derived neurotrophic factor (pBDNF) by an average of 59% (range 30–87%; p<0.05). When normalised for duration at target power, SupraHIIT produced a 5–10-fold greater increase than MICT. Both modalities lead to a variable response in other exerkines including clusterin, lactate, hepatocyte growth factor and interleukin-6.

**Conclusion:**

In COPD, short-duration SupraHIIT is more feasible and enables markedly higher external exercise intensities than MICT. By elevating pBDNF and other potentially beneficial exerkines, it shows potential for extrapulmonary benefits.

## Shareable abstract

**Short-duration, supramaximal HIIT is a feasible exercise modality in people with COPD. It enables high exercise intensities without excessive dyspnoea and supports the release of neurotrophic factors that can benefit cognitive function.** https://bit.ly/3DSzYvG

## Graphical abstract

**Figure:**
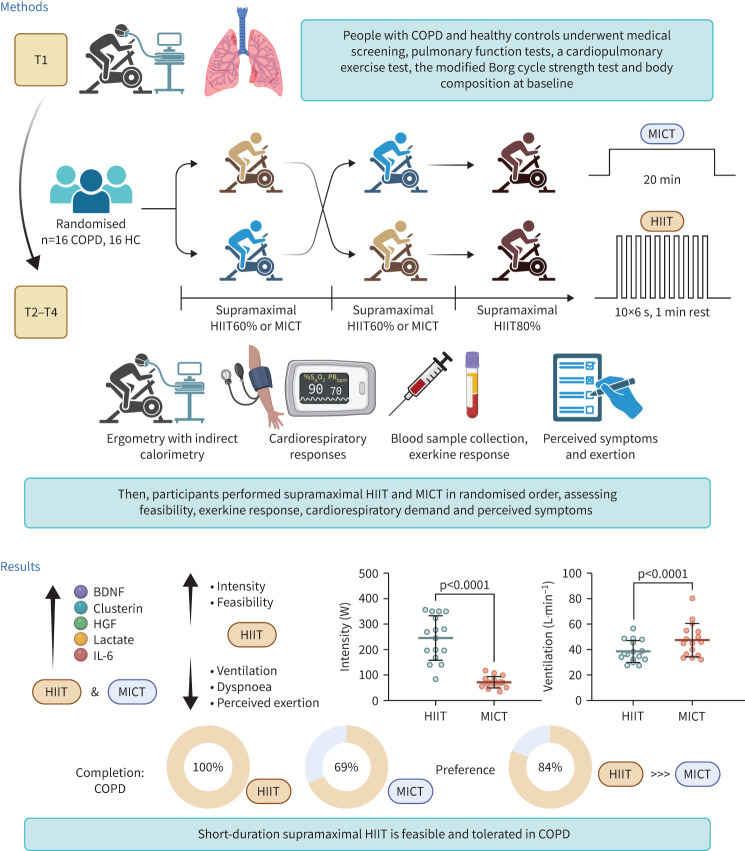
An overview of the study design and the results. HC: healthy control; HIIT: high-intensity interval training; supramaximal HIIT60%: external intensity at 60% of maximum mean power output for 6 s; supramaximal HIIT80%: external intensity at 80% of maximum mean power output for 6 s; MICT: moderate-intensity continuous training; BDNF: brain-derived neurotrophic factor; HGF: hepatocyte growth factor; IL-6: interleukin-6; T1: time point 1; T2–T4: time points 2, 3 and 4.

## Introduction

COPD is closely associated with systemic manifestations beyond the lungs. These include impaired brain health [1], reduced cardiovascular fitness, cardiometabolic disease [2, 3] and reduced limb muscle function [4] among others. Individuals with COPD face a four-fold higher risk of cognitive impairment than those without the disease [5], impairing medication adherence and inhaler technique [6, 7], leading to increased hospitalisations. Regardless of lung function impairment, extrapulmonary manifestations are associated with reduced physical function, lower quality of life and a multiple-fold increase in mortality risk [1, 4]. As a key component of pulmonary rehabilitation [8], exercise training may help counteract these extrapulmonary manifestations in COPD.

Exercise promotes brain health by stimulating exerkines such as brain-derived neurotrophic factor (BDNF) [9, 10], a key mediator for neurocognitive benefits [11, 12]. In healthy individuals, exercise intensity correlates with a transient increase in BDNF levels in the blood, with the highest levels observed following high-intensity exercise [9]. High-intensity exercise is also a key factor for activation of peroxisome proliferator-activated receptor γ coactivator (PGC)-1α, an important regulator of mitochondrial biogenesis [13]. In turn, upregulation of PGC-1α is key for stimulating production of BDNF and pathways for neuroprotection [14]. Consequently, high-intensity interval-training (HIIT) is recommended for cognitive benefits [9, 15]. It is also used to improve cardiovascular fitness and muscle function, as it can confer similar or superior physiological adaptations compared with moderate-intensity continuous training (MICT) in a wide-range of populations [16, 17]. However, achieving high exercise intensities is challenging for people with COPD due to ventilatory limitations and impaired pulmonary mechanics [18], which precipitate intolerable dyspnoea [19].

In COPD, HIIT improves exercise tolerance, quality of life and skeletal muscle properties as effectively as continuous exercise but with less time commitment, ventilatory demand and dyspnoea [20–22]. Subsequently, HIIT has gained interest in pulmonary rehabilitation [21, 23]. Previous studies typically utilised intensities of 70–130% of maximal aerobic power (MAP), defined as the highest workload reached during a cardiopulmonary exercise test (CPET). Work-to-rest ratios varying from 20:40 s to 4:4 min have been used [24]. Although these intensities exceed those of MICT, studies in healthy populations suggest that supramaximal intensities, which exceed MAP, can reach 200–300% of MAP with adjusted work-to-rest ratios or exercise durations [25, 26].

Specifically, short-duration supramaximal HIIT (SupraHIIT) using a 6:54 s work-to-rest ratio has proven feasible and well tolerated in older inactive adults, achieving external exercise intensities close to 300% of MAP [25–27]. Using individualised intensity, rather than an all-out effort, makes is manageable for untrained individuals [25]. While yet to be explored in COPD, its short intervals and low work-to-rest ratio, make it promising for this population. SupraHIIT can elicit high metabolic stress [28], elevate BDNF and other exerkines [29], and yield improvements in cardiovascular fitness and other health markers [26].

In this study, we evaluated the feasibility and acute physiological responses of this novel SupraHIIT protocol. We hypothesised that SupraHIIT would be feasible, allowing higher exercise intensities than MICT with a lower ventilatory demand. Additionally, we hypothesised that the higher exercise intensities of SupraHIIT would elicit a greater increase in plasma BDNF (pBDNF). Matched healthy controls (HCs) were included to elucidate the impact of COPD on exercise responses.

## Methods

Additional details are found in the supplementary materials.

### Trial design

**FIGURE 1:**
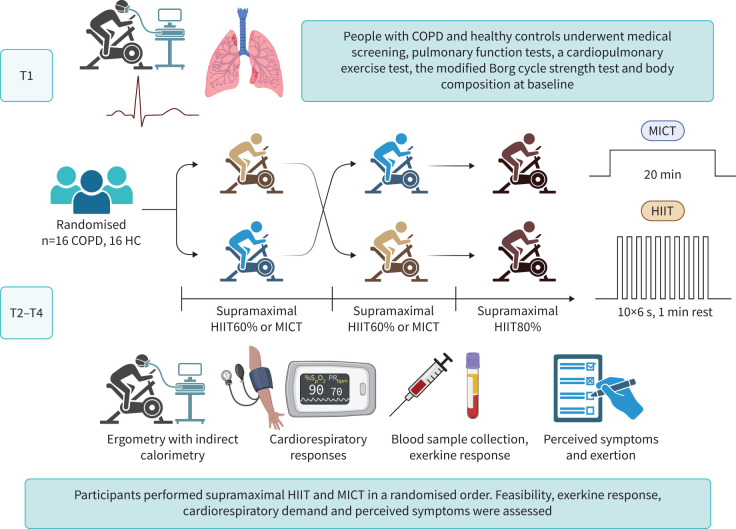
Overall design of the randomised crossover trial using an ABC/BAC sequence. Each of the four occasions was interspersed with at least 48 h of rest and participants completed the trial within 2 weeks. Pulmonary function and cardiopulmonary exercise tests were performed at the Unit of Clinical Physiology at University Hospital of Umeå, while the remaining assessment was performed at Umeå Movement and EXercise laboratory at Umeå University (UMEX). Participants were instructed to refrain from vigorous physical activity 48 h before each visit. In addition, they were instructed to avoid caffeine intake and smoking 6 and 8 h before each visit, respectively, while continuing to adhere to their regular medication routines. To control for circadian fluctuations on performance and physiological effects, visits 2, 3 and 4 were scheduled at the same time of day (09:00 or 13:00). Supramaximal HIIT60% and 80% refers to the external intensity being the percentage of maximum mean power output for 6 s. T1: visit 1 (baseline), T2–T4: visit 2 to 4. HC: healthy control; HIIT: high-intensity interval training; MICT: moderate-intensity continuous training.

**FIGURE 2:**
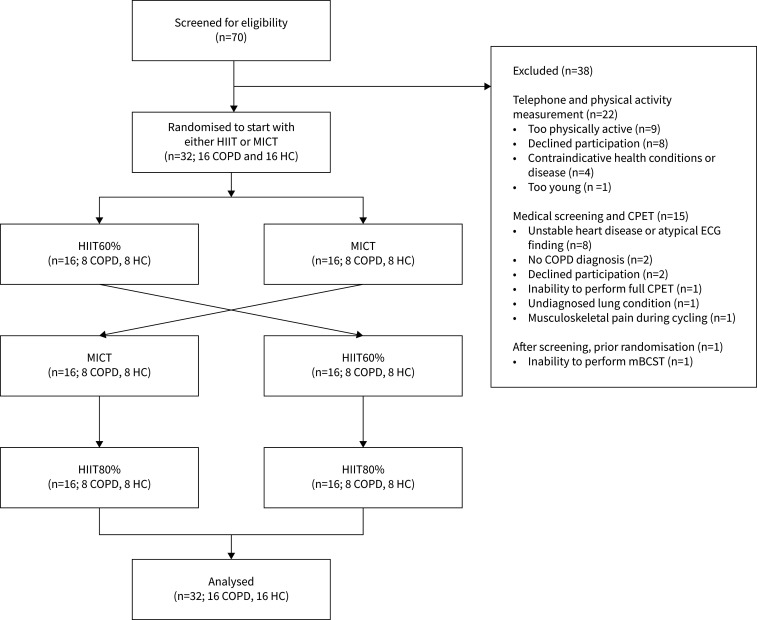
CONSORT flowchart. HC: healthy control; HIIT: supramaximal high-intensity interval training; HIIT60%: supramaximal HIIT at 60% of maximum mean power output for 6 s; HIIT80%: supramaximal HIIT at 80% of maximum mean power output for 6 s; MICT: moderate-intensity continuous training; mBCST: modified Borg cycle strength test; CPET: cardiopulmonary exercise test.

Between March and December 2022, we conducted a single-centre, randomised controlled crossover trial at Umeå University, Sweden (ClinicalTrials.gov: NCT05874999). The trial adhered to the Declaration of Helsinki and was approved by the Swedish Ethical Review authority (no. 2021-05408-02). All participants provided written informed consent. The trial design is outlined in figure 1, and the CONSORT flowchart in figure 2. Reporting of this trial follows applicable items from the CONSORT extension for crossover trials [30] and Consensus on Exercise Reporting Template [31].

### Participants

We used a convenience sampling method to include participants of both sexes aged ≥60 years. Participants with COPD were eligible if they had a diagnosis consistent with Global Initiative for Chronic Obstructive Lung Disease criteria [32], while HCs had normal pulmonary function. Exclusion criteria included conditions making exercise unsafe (*e.g.* unstable heart disease), a COPD exacerbation in the past 6 weeks, and for HCs, a physical activity level too low or high for matching (further details in supplementary materials).

### Procedures

The first visit encompassed pulmonary function tests, including spirometry, whole-body plethysmography and single-breath diffusion capacity of the lung for carbon monoxide (*D*_LCO_) [33, 34] (Vyntus ONE, Vyaire Medical Inc., IL, USA). Then, a CPET was performed following European Respiratory Society guidelines [35]. After 1.5 h of recovery, participants completed a modified Borg cycle strength test (mBCST) on a Monark LC6 (Monark, Vansbro, Sweden). The mBCST is a submaximal cycling test suitable in older adults to prescribe HIIT intensities [27]. It estimates the maximum mean power output (MPO) over 30 s without requiring maximal effort. The test consists of 30-s bouts at 80–90 revolutions per min (rpm), with increasing intensity, interspersed with 30-s recovery periods, and continues until the participant reaches a Borg rating of perceived exertion (RPE) of ≥17 or is unable to maintain the intended cadence. We adjusted the stage increments to our population, based on participants’ MAP, to range from 15 to 50 W. Then, the MPO output for 6 s (MPO_6_) was calculated by multiplying the highest achieved workload by 1.75 [27], as successfully applied in healthy elderly individuals [26]. Additional baseline characteristics measurements, obtained during the first visit, are detailed in the supplementary materials.

### Randomisation

The second and third visit included SupraHIIT and MICT in a randomised order. Participants were allocated in a 1:1 ratio using a random sequence stratified by sex, which was computer-generated using a randomisation tool (randomization.com) by the principal investigator. Assessors were not blinded and allocation was not concealed. Participants were informed of the study's general purpose but not its hypotheses.

### Exercise protocol

All exercise sessions began with a 5-min warm-up and ended with a 5-min cool-down at 30% of MAP. The first supramaximal HIIT was performed as ten 6-s intervals at 80–90 rpm, interspersed with a 54-s recovery, consisting of 24 s of passive rest followed by 30 s of active recovery at 30% of MAP (supplementary figure S1). Interval intensity was individualised at 60% of MPO_6_ (called HIIT60%), corresponding to approximately 150% of each participant's MAP, though this may vary slightly depending on performance on the CPET *versus* mBCST. The total duration of the session was 20 min. Our approach of SupraHIIT closely followed the methods descibred by Hedlund *et al.* [27] and Simonsson *et al.* [26], with slight modifications tailored to the current cycle ergometer setup.

The MICT was performed at 60% of MAP for 20 min at 60–70 rpm, following pulmonary rehabilitation guidelines [8], with a session duration of 30 min (supplementary figure S1). The fourth visit encompassed SupraHIIT at 80% of MPO_6_ (called HIIT80%), corresponding to approximately 200% of MAP. This session was predetermined as the last session to ensure an unbiased comparison between HIIT60% and MICT, not knowing the feasibility *a priori*. Sessions were performed individually supervised by a physiotherapist or exercise physiologist. As we aimed to investigate the feasibility and physiological response to the exercise modalities performed as they would typically be applied in a real-world setting, the modalities were not matched for total work.

### Measurements

During exercise sessions, we measured respiratory gas exchange breath by breath with a Metamax-3B (Cortex BioPhysik, Leipzig, Germany [36]), blood oxygen saturation (Nonin 3150, Nonin Medical, Plymouth, MN, USA), blood pressure (Tango M2, SunTech Medical, Inc., FL, USA) and heart rate ((HR), Polar H9, Polar Electro Oy, Kempele, Finland), perceived exertion (Borg RPE [37]), dyspnoea and leg fatigue (Borg CR-10 [38]), as described in supplementary materials. In addition, we asked which exercise modality participants preferred.

Blood samples were collected at rest, during and after exercise. Analyses were performed in triplicate by a blinded technician using single- or multiplex fluorescent bead-based immunoassays [39] (Luminex Assay, Luminex Corporation, TX, USA) using a Bio-Plex 200 (Bio-Rad Laboratories Inc., CA, USA) or ELISA [40] using a Synergy HT (Agilent Technologies Inc., CA, USA), following the manufacturer's protocols with commercially available kits (see supplementary table S1 for details).

### Outcomes

Primary outcomes were external exercise intensity (W) and change from baseline in circulating pBDNF (pg·mL^−1^, also expressed as % change from baseline and change per min of exercise at target intensity) during exercise. Plasma BDNF is a well-established exerkine with neurocognitive benefits. Specifically, we were primarily interested in pBDNF, as it is likely of greater physiological importance than serum BDNF (sBDNF), as discussed by others [15, 41].

Secondary outcomes included cardiorespiratory parameters (mean during exercise, iso-time and end of exercise), symptoms and RPE at multiple time points, feasibility of the modalities, change-from-baseline exerkines (absolute and % change) and safety outcomes, as further detailed in supplementary materials. Iso-time comparisons were defined as at the end of SupraHIIT compared with 10-min of MICT.

### Statistical analysis

Continuous variables were reported as mean±sd for normally distributed data, as determined by Q–Q plots and histograms, if not mentioned otherwise. Non-normally distributed data were presented as median (interquartile range). Differences in exercise intensity, cardiorespiratory outcomes, symptoms, exerkines concentrations and total work performed were assessed using a one-way repeated measures (RM) ANOVA with participants as random effects. Tukey's test was used for multiple comparisons. Log transformations were applied as needed to meet model assumptions. Pairwise comparisons were analysed with an independent t-test, Welch's test, Mann–Whitney U-test or Fisher's exact test. Quade's test with daylight time as a covariate was used for physical activity analysis. We used Pearsons and Spearman's correlations for exploratory analyses investigating associations of baseline characteristics and selected outcomes. Statistical analyses were conducted using JMP Pro v.17.2 with a two-sided α level of 0.05.

Sample size was calculated to detect a mean difference in exercise intensity of 50±30 W [9] with an α level of 0.05 and 80% power, considered the smallest difference of interest. This revealed a minimum sample size of seven. Regarding pBDNF, a sample size of 16 was based on the mean sample size in two meta-analyses examining the acute response of exercise on BDNF. These found an effect size ranging from 0.99 to 1.10 for pBDNF [15, 42], which reveals a sample size of 10. Conservatively, a sample size of 16 can detect an effect size of 0.8. The sample size was also judged as reasonable to investigate our feasibility aims [43] and other secondary outcomes.

## Results

32 individuals were included (figure 2) and their baseline characteristics are shown in table 1.

**TABLE 1:** Baseline characteristics of the participants.

	COPD (n=16)	HC (n=16)	p-value
**Anthropometrics**			
Age, years	74.8±6.1	74.2±4.5	0.746
Female sex	8 (50)	8 (50)	
Height, cm	169.4±7.3	171.4±7.3	0.459
Weight, kg	71.9±14.8	74.0±7.2	0.617
BMI, kg·m^−2^	24.9±3.8	25.2±2.1	0.782
FFMI, kg·m^−2^	17.4±2.6	17.9±1.6	0.511
Fat mass, %	29.9±8.8	28.7±7.8	0.689
**Cardiorespiratory fitness**			
MAP, W	118.8±37.4	163.8±38.1	**0.002**
MAP, % predicted^#^	85±20	116±22	**<0.001**
*V̇*_O_2__peak (mL·kg^−1^min^−1^)^¶^	21.8±5.2	25.4±3.8	**0.003**
BCST Wpeak, W	175±63	256±55	**<0.001**
Number of steps per day, unadjusted^+^	6727±3212	8450±2520	0.069
Number of steps per day, daylight adjusted^+^	5458 (4040–9726)	8266 (6145–11 852)	0.055
**Clinical characteristics**			
CAT score, 0–40	14.2 (5.7)	NA	
mMRC dyspnoea score, 0–4, n	2/7/5/1/1	6/10/0/0/0	**0.022**
GOLD stage, I/II/III/IV	5 (31)/10 (63)/1 (6)/0 (0)	NA	
GOLD stage A/B/E^§^	4 (25)/12 (75)/0 (0)	NA	
**Exacerbations and hospitalisations last 12 months, 0, 1 and ≥2**			
Exacerbations	15 (94), 1 (6), 0 (0)	NA	
Hospitalisations	13 (81), 1 (6), 2 (13)	16 (100), 0 (0), 0 (0)	0.226
AECOPD hospitalisations	16 (100), 0 (0), 0 (0)	NA	
Nonrespiratory hospitalisations	13 (81), 2 (13), 1 (6)	16 (100), 0 (0), 0 (0)	0.226
Respiratory hospitalisations	15 (94), 1 (6), 0 (0)	16 (100), 0 (0), 0 (0)	1.000
Emergency visits	10 (63), 4 (24), 2 (13)	13 (81), 2 (13), 1 (6)	0.528
**Smoking**			
Smoking status			**0.006**
Current smoker	1 (6)	0 (0)	
Ex-smoker	14 (88)	7 (44)	
Never-smoker	1 (6)	9 (56)	
Pack-years	24.2±16.6	5.4±9.8	**<0.001**
**Medications**			
Noninhaler medications	4.4 (2.7)	1.6 (1.5)	**0.002**
Inhaled medications	2 (1–3)	0 (0–0)	**<0.001**
ICS use	6 (38)	1 (6)	
SABA only	1 (6)	0 (0)	
LAMA	14 (88)	0 (0)	
LABA+LAMA	12 (75)	0 (0)	
LABA+LAMA+ICS	5 (31)	0 (0)	
Oxygen therapy	0 (0)	0 (0)	
Cholesterol lowering	5 (31)	5 (31)	1.000
Beta blockers	7 (44)	2 (13)	0.113
Other heart medication	12 (75)	8 (50)	0.273
**Comorbidities** ^f^			
Any	16 (100)	12 (75)	**0.013**
Hypertension	9 (56)	8 (50)	0.723
Hyperlipidaemia	5 (31)	5 (31)	0.694
**Lung function parameters**			
**Spirometry**			
FEV_1_, L	1.8±0.3	2.8±0.5	**<0.001**
FEV_1_, % predicted	73±13	106±13	**<0.001**
FVC, L	3.3±0.7	3.8±0.7	0.069
FVC, % predicted	98±16	108±12	**0.044**
FEV_1_/FVC	0.56±0.08	0.73±0.04	**<0.001**
IC, L	2.5±0.5	2.8±0.5	0.096
MVV, L·min^−1^	69.6±14.1	92.9±21.4	**0.001**
**Diffusion capacity**			
*D*_LCO_ SB, mmol·min^−1^·kPa^−1^	5.2±1.5	6.8±1.3	**0.004**
*D*_LCO_ SB, % predicted	72±16	90±16	**0.003**
**Lung volumes**			
TLC, L	6.0±1.1	6.3±1.3	0.562
TLC, % predicted	97±11	97±7	0.922
FRC, L	3.9±0.9	3.7±1.1	0.522
FRC, % predicted	105±27	93±12	0.103
FRC/TLC, %	65±6	58±7	**0.006**
RV, L	2.6±0.5	2.3±0.6	0.263
RV, % predicted	98±26	84±14	0.078
RV/TLC, %	43±4	37±5	**0.002**
**Blood markers** ^##^			
Plasma BDNF, pg·mL^−1^	3638±1762	3246±1745	0.786
Serum BDNF, pg·mL^−1^	22 529±6173	23 522±5962	0.647
Clusterin, ng·mL^−1^	173 570±34 192	176 456±25 845	0.586
Irisin, ng·mL^−1^¶¶^^	8.3±1.9	9.0±2.0	0.345
Cathepsin B^++^, ng·mL^−1^	157±79	181±94	0.476
Lactate, mM	1.06±0.27	0.96±0.21	0.239
VEGF, pg·mL^−1^	14.3 (7.2–25.2)	13.5 (11.0–20.7)	0.895
HGF, pg·mL^−1^	131 (91–174)	119 (91–173)	0.955
Interleukin-6, pg·mL^−1^	1.05 (0.77–1.24)	0.60 (0.46–1.08)	**0.021**
Interleukin-8, pg·mL^−1^	4.59±2.56	3.84±1.33	0.311
Interleukin-10, pg·mL^−1^	0.51 (0.33–0.86)	0.41 (0.29–0.54)	0.207
TNF, pg·mL^−1^	8.52±2.26	8.29±3.31	0.819

Data are presented as mean±sd, median (IQR) or n (%). n=16 for all except otherwise stated. HC: healthy control; BMI: body mass index; FFMI: fat-free mass index; AECOPD: acute exacerbation of COPD; CAT: COPD Assessment Test; mMRC: modified Medical Research Council; GOLD: Global Initiative for Chronic Obstructive Lung Disease; ICS: inhaled corticosteroid; SABA: short-acting β-agonist; LAMA: long-acting muscarinic antagonist; LABA: long-acting β-agonist; FEV_1_: forced expiratory volume in 1 s; FVC: forced vital capacity; IC: inspiratory capacity; TLC: total lung capacity; FRC: functional residual capacity; RV: residual volume; *D*_LCO_ SB: diffusion capacity of the lung for carbon monoxide – single breath; BDNF: brain-derived neurotrophic factor; VEGF: vascular endothelial growth factor; HGF: hepatocyte growth factor; TNF: tumour necrosis factor; NA: not applicable. ^#^: Using Brudin 2014 equation. ^¶^: Using Gläser 2013 equation, ^+^: n=15 for HC due to missing data, ^§^: CAT score used as instrument to determine GOLD ABE category. ^f^: Selection of the most common comorbidities, ^##^: mean value from the three baseline samples taken before each exercise sessions. ^¶¶^: n=13 for COPD, n=15 for HC. ^++^: n=14 for both groups.

### Primary outcomes

#### Exercise intensity

**FIGURE 3:**
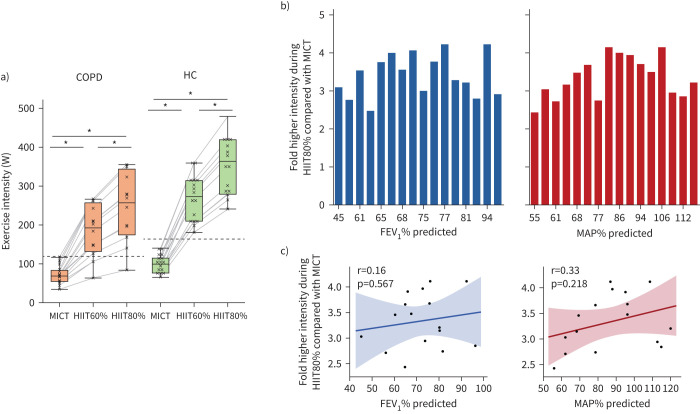
Supramaximal high-intensity interval training (HIIT) enabled a 2–3.5-fold increase in exercise intensity compared with moderate-intensity continuous training (MICT), independent of airflow obstruction and predicted exercise capacity. a) Boxplots of external exercise intensity (W) during each modality in COPD (left) and healthy controls (HCs) (right). Dashed line indicates the mean maximal aerobic power (MAP) for each group. *: Significant difference between modalities, Tukey's *post hoc* test (p<0.05). b) In participants with COPD, we explored the association between airflow obstruction, exercise capacity and the ability to increase intensity from MICT to supramaximal HIIT. As seen, the relative increase in exercise intensity occurs independent of the degree of airflow obstruction (forced expiratory volume in 1 s (FEV_1_) % predicted) or exercise capacity (MAP % predicted). Each bar represents a participant with COPD, sorted from low to high FEV_1_ % predicted (left, in blue) and MAP % predicted (right, in red). The fold increase in intensity relates to supramaximal HIIT at 80% of maximum mean power output for 6 s compared with MICT at 60% of MAP derived from a cardiopulmonary exercise test. c) As indicated by the correlation coefficient shown, there was no linear correlation between FEV_1_ % predicted (left) or MAP % predicted and the increase in intensity in people with COPD (n=16). For HCs, comparable results were seen (not shown in figure): (r(16)=−0.35, p=0.194 for FEV_1_ % predicted, r(16)=−0.04, p=0.878 for MAP % predicted).

In COPD participants, external exercise intensity during HIIT60% was 113 W higher and during HIIT80%, 174 W higher than during MICT, corresponding to 2.0- and 3.5-fold increases, respectively (figure 3a). Comparable results were observed in HCs (figure 3a and table 2). Exploratory analyses found no significant correlation between forced expiratory volume in 1 s (FEV_1_) % predicted or MAP % predicted and the increase in exercise intensity from MICT to SupraHIIT in COPD participants (figure 3b,c), suggesting that the relative increase in exercise intensity is consistent, regardless of airflow obstruction or baseline exercise capacity.

**TABLE 2:** Exercise characteristics, cardiorespiratory parameters, perceived exertion and symptoms during supramaximal HIIT and MICT.

	HIIT60%			HIIT80%			MICT		
	COPD	HC	p-group	COPD	HC	p-group	COPD	HC	p-group
**Exercise intensities**									
Intensity, W	184±66^#¶^	269±58^#¶^	**<0.001**	245±88^#^	358±77^#^	**<0.001**	71±22	98±23	**0.002**
Intensity, W, min–max	63–267	181–360		84–356	241–480		34–117	65–140	
Intensity, % MAP	153±25^#¶^	165±17^#¶^	0.119	204±33^#^	220±23^#^	0.114	60±0	60±0	
Intensity, % MICT	255±42^¶^	276±29^¶^	0.115	340±56	367±39	0.116			
**Cardiorespiratory parameters**									
*V*′_O_2__, L·min^−1^	1.0±0.2^#¶^	1.3±0.2^#¶^	**<0.001**	1.1±0.2^#^	1.4±0.3^#^	**0.007**	1.3±0.3	1.6±0.3	**0.006**
*V*′_O_2__, mL·kg^−1·^min^−1^	14.5±2.7^#¶^	17.7±2.2^#¶^	**0.001**	16.1±3.3^#^	18.8±3.2^#^	**0.027**	18.6±3.5	21.8±3.3	**0.012**
*V*′_O_2__, % peak	68±11^#¶^	70±6^#¶^	0.641	75±11^#^	74±14^#^	0.930	87±11	86±10	0.914
HR, bpm	99±15^#^	108±11^#¶^	0.070	102±13^#^	116±15^#^	**0.011**	111±17	128±12	**0.003**
HR, % peak	72±8^#^	71±5^#¶^	0.610	74±7^#^	77±7	0.163	80±9	85±5	0.054
RER, ratio	0.90±0.02^#^	0.91±0.04^¶^	0.443	0.92±0.04	0.94±0.03	0.154	0.93±0.04	0.93±0.02	0.812
*V*′_E_, L	38.5±8.6^#^	37.8±8.2^#¶^	0.819	41.7±9.6^#^	43.7±8.2^#^	0.546	47.4±13.1	48.6±10	0.774
*V*′_E_/MVV, %	56±14^#^	38±9^#¶^	**<0.001**	61±15^#^	44±7^#^	**<0.001**	70±17	49±10	**<0.001**
*V*′_E_/*V*′_O_2__	33.1±5.4	26.1±3.4^¶^	**<0.001**	33.2±5.6	29±4.3^#^	**0.026**	32.9±5.7	27.8±4.5	**0.009**
*V*′_E_/*V*′_CO_2__	36.9±5.8	28.9±3.8^¶^	**<0.001**	36.0±6.8	30.8±4.2	**0.014**	35.4±6	30.0±4.5	**0.007**
BF, min^−1^	28.3±4.6^#^	24.5±3^#¶^	**0.011**	28.5±3.8^#^	26.3±3.3	0.089	30.5±5.5	27.4±4.3	0.090
*V*_T_, L	1.4±0.3^#¶^	1.6±0.2^#¶^	0.117	1.5±0.4	1.7±0.3^#^	0.132	1.6±0.4	1.8±0.3	0.117
*V*′_CO_2__, L·min^−1^	0.94±0.17^#¶^	1.19±0.2^#¶^	**0.001**	1.05±0.23^#^	1.31±0.25^#^	**0.005**	1.22±0.29	1.5±0.29	**0.009**
*S*_pO_2__, %	95±2^#^	95±1^#^	0.568	94±2^#^	95±2	0.449	93±2	94±1	0.181
SBP, mmHg	160±29^#^	156±29^#^	0.678	152±37^#^	155±27^#^	0.819	182±32	187±23	0.637
DBP, mmHg	79±15	74±9	0.277	75±13	71±13	0.451	78±12	71±11	0.079
Mean AP, mmHg	106±17	101±10^#^	0.365	101±18^#^	99±14^#^	0.802	113±14	110±11	0.461
**Perceived exertion and symptoms, mean during exercise**									
RPE, points	13.5±2.4^#^	12.5±2.6	0.267	14.3±2.0	13.3±2.2	0.170	15.1±2.3	13.1±2.0	**0.014**
Dyspnoea, points	4.3±1.8^#^	3.0±1.5^#^	**0.034**	5.0±1.5	3.6±1.3	**0.006**	5.5±1.7	3.7±1.3	**0.002**
Leg fatigue, points	4.1±1.7^#^	3.0±1.5^#^	**0.044**	4.9±1.5	3.4±1.7	**0.014**	5.4±1.8	3.7±1.5	**0.008**
Session RPE, points	3.9±1.6^#^	3.2±1.3^#^	0.149	4.6±1.8	3.6±1.4^#^	0.117	5.3±1.9	4.9±2.1	0.595

Exercise intensities, cardiorespiratory parameters, blood pressure, perceived exertion and symptoms during exercise. Data are shown as mean±sd if not otherwise mentioned. Indirect calorimetry data, blood pressure and perceived exertion/symptoms are averages over the whole exercise session (10 min of supramaximal HIIT, up to 20 min of MICT), excluding warm-up and cool-down. Selected variables (*V′*_O_2__ (% peak), HR (% peak), RER and dyspnoea are depicted in supplementary figure S2. n=16 for all except HC HIIT60% where n=14 for *V′*_O_2__/*V′*_CO_2__-related variables and n=15 for *V′*_E_-related variables due to technical issues. Supramaximal HIIT at 60% (HIIT60%) and 80% (HIIT80%) are performed at 60% and 80%, respectively, of maximum mean power output for 6 s. MICT is performed at 60% of maximal aerobic power. HC: healthy control; MAP: maximal aerobic power; *V′*_O_2__: oxygen consumption; *V′*_CO_2__: carbon dioxide production; HR: heart rate; RER: respiratory exchange rate; *V′*_E_: minute ventilation; MVV: maximal voluntary ventilation; BF: breathing frequency; *V*_T_: tidal volume; *S*_pO_2__: peripheral oxygen saturation; SBP: systolic blood pressure; DBP: diastolic blood pressure; AP; arterial pressure; RPE: rating of perceived exertion. Tukey's *post hoc* comparisons were made after a significant main effect: ^#^: significantly different from MICT (p<0.05), ^¶^: significantly different from HIIT80 (p<0.05). p-group indicates p-value for pairwise comparison with independent t-test or Mann–Whitney U-test.

#### Brain-derived neurotrophic factor

**FIGURE 4:**
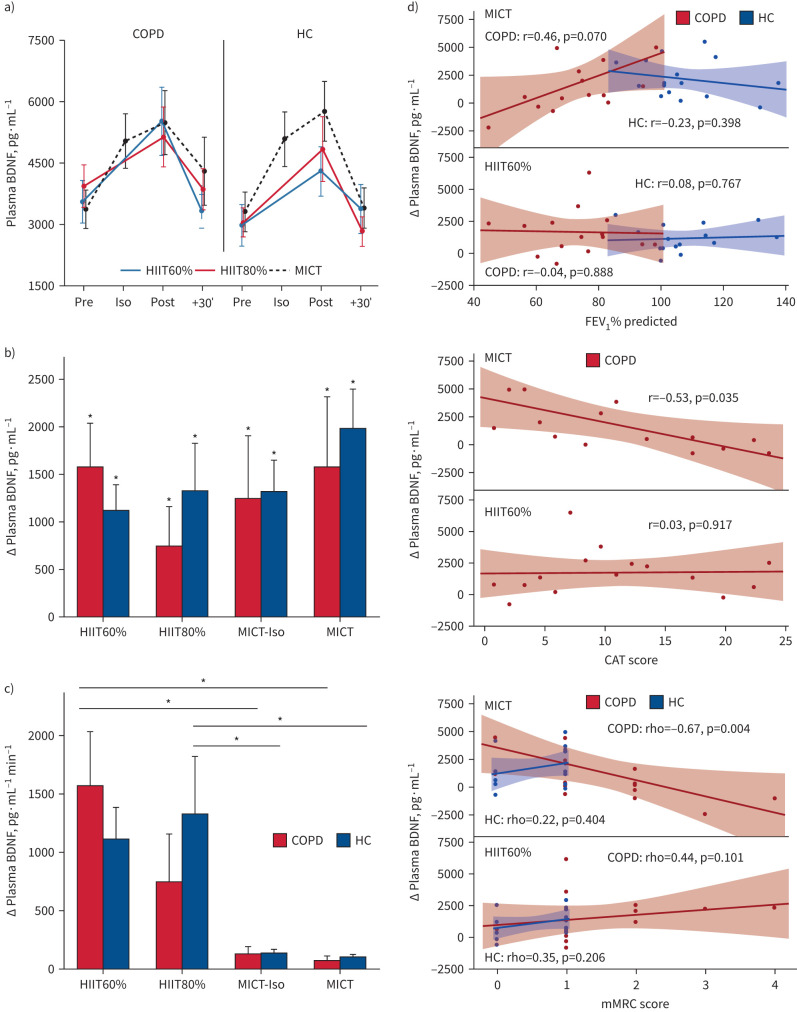
Exercise-induced change in plasma brain-derived neurotrophic factor (pBDNF). Data are shown as mean±se (a, b and c). For people with COPD and healthy controls (HCs), pBDNF increased in both supramaximal high-intensity interval training (HIIT) and moderate-intensity continuous training (MICT). a) pBDNF increased in all sessions in both groups (supplementary tables S2 and S3). Independent t-tests showed no group-difference in pBDNF at any time point (p<0.05 for all). b) Change from baseline in pBDNF for each session depicting a significant increase (p<0.05 for all, supplementary table S2) from baseline to immediately after all sessions, as well as to MICT-Iso (10 min of MICT). There was no difference in change between the modalities or between groups revealing that those with COPD had a similar increase as HCs (also see supplementary tables S2 and S3 and supplementary figure S1). c) Change from baseline in pBDNF adjusted for time spent at target intensity (60 s (10×6 s) for supramaximal HIIT, up to 20 min for MICT). d) To explore whether the change in pBDNF exercise was related to airflow obstruction (forced expiratory volume in 1 s (FEV_1_) % predicted, upper right), impact of COPD (CAT, middle right) or dyspnoea (modified Medical Research Council (mMRC) score, lower right) we correlated the change in pBDNF to these variables. For FEV_1_ % predicted, there was no significant Pearson correlation in supramaximal HIIT (HIIT60%: r(15)=−0.04, p=0.888; HIIT80%: r(15)=0.26, p=0.350), whereas a nonsignificant moderate correlation of reduced change in pBDNF with lower FEV_1_ % predicted was seen in MICT: r(16)=0.46, p=0.070). No correlations were seen for HCs regarding change in pBDNF and FEV_1_ % predicted (HIIT60%: r(15)=0.08, p=0.767; HIIT80%: r(15)=−0.10, p=0.712; MICT: r(16)=−0.23, p=0.398) or MAP % predicted (HIIT60%: r(15)=0.04, p=0.876; HIIT80%: r(15)=−0.01, p=0.960; MICT: r(16)=0.02, p=0.932). Shaded area represents the 95% confidence interval of the correlation coefficient. HIIT60%: supramaximal HIIT at 60% of maximum mean power output for 6 s; HIIT80: supramaximal HIIT at 80% of maximum mean power output for 6  s. *: Significant difference (p<0.05) for change from baseline (c) or between modalities (d), Tukey's *post hoc* test.

Circulating pBDNF levels significantly increased during both SupraHIIT and MICT, with no differences between the exercise modalities or between COPD and HC (figure 4a,b and supplementary tables S2 and S3). On average, the relative increase was 59% (range 30–87%; supplementary table S3 and figure S2). When normalised per min of exercise at target power, SupraHIIT produced a 5–10-fold greater increase compared with MICT, although this difference was not consistently statistically significant (figure 4c and supplementary table S4).

Exploratory analysis revealed no correlation between changes in pBDNF and FEV_1_ % predicted, COPD Assessment Test (CAT) or modified Medical Research Council (mMRC) during SupraHIIT in participants with COPD (figure 4d). Contrastingly, for MICT, both CAT and mMRC was negatively strongly correlated with change in pBDNF, while a moderate nonsignificant correlation was observed for FEV_1_ % predicted (figure 4d). Notably, a significant strong correlation between change in pBDNF and MAP % predicted for MICT was seen (r(16)=0.51; p=0.042) but not for HIIT60% or HIIT80% (r(15)=−0.24, p=0.389; r(15)=0.30, p=0.282, respectively). In HCs, FEV_1_ % predicted, mMRC or MAP % predicted was not correlated with the change in pBDNF (figure 4).

### Secondary outcomes

#### Feasibility

SupraHIIT, both HIIT60% and HIIT80% was feasible, with a 100% completion rate in both groups. In contrast, 5 of 16 COPD participants could not complete the 20 min of MICT (range 5–20 min; p=0.008), while all HCs could (p=0.043 between groups). Supramaximal HIIT showed high fidelity, requiring no modifications to intensity, cadence or duration. As shown, RPE and symptoms were lower in SupraHIIT than in MICT, resulting in reduced ventilatory demand (table 2 and supplementary tables S5 and S6). Notably, 13 of 16 COPD participants (p=0.009) and 14 of 16 HCs (p=0.002) preferred SupraHIIT to MICT.

#### Cardiorespiratory demand, perceived exertion, dyspnoea and leg fatigue

**FIGURE 5:**
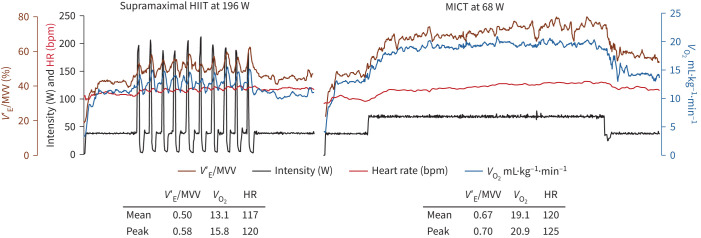
An overview of a session of supramaximal high-intensity interval training (HIIT) (left) and moderate-intensity continuous training (MICT) session (right) in a representative participant with COPD who could complete the MICT session. The intervals in the supramaximal HIIT session are performed at 80% of maximum power output for 6 s, resulting in 196 W. MICT is performed at 60% of maximal aerobic power (MAP), resulting in 68 W. As seen, the ventilatory demand was higher in MICT as demonstrated by the higher mean and peak minute ventilation (*V*′_E_)/maximal voluntary ventilation (MVV) ratio. *V*_O_2__: oxygen consumption.

As hypothesised, the ventilatory demand in SupraHIIT was reduced compared with MICT, as demonstrated by the minute ventilation (*V′*_E_), *V′*_E_/maximal voluntary ventilation (MVV) ratio and breathing frequency (table 2, supplementary table S5 and supplementary figure S3). During SupraHIIT, participants with COPD had a mean % oxygen consumption (*V′*_O_2__) peak of 68% and 75% (HIIT60% and HIIT80%, respectively), reaching 77% and 85% at end of exercise (supplementary table S5 and supplementary figure S3). Regarding HR, they exercised at 72–74% and 77–80% of % HRpeak, respectively. Similar values were seen for HCs. In both groups, MICT induced a higher *V′*_O_2__ and HR (table 2 and supplementary table S5). Figure 5 depicts an overview of a SupraHIIT and MICT session in a representative participant with COPD.

Total work performed was significantly lower in both SupraHIIT sessions compared with MICT (p<0.001) in both COPD and HC but did not differ between the two SupraHIIT modalities. In COPD, total workload was 51±17 kJ (HIIT60%), 56±18 kJ (HIIT80%) and 110±35 kJ (MICT), with MICT requiring 2.2±0.2 and 2.0±0.2-times more work than HIIT60% and HIIT80%, respectively. In HCs, workloads were 72±16 kJ (HIIT60%), 78±17 kJ (HIIT80%) and 151±35 kJ (MICT), corresponding to 2.1±0.1 and 1.9±0.1-times higher work in MICT than SupraHIIT. HCs produced more work compared with those with COPD in all sessions (p<0.001).

During all sessions, those with COPD had a lower *V′*_O_2__ compared with HCs. In relation to *V′*_O_2__peak and HRpeak, those with COPD exercised at a similar relative intensity as HCs. The peripheral oxygen saturation (*S*_pO_2__) and blood pressure response were similar between individuals with COPD and HCs across all sessions. During SupraHIIT, both groups exhibited lower systolic blood pressure compared with MICT and those with COPD had a higher *S*_pO_2__ (table 2). Among people with COPD, clinically relevant reductions in dyspnoea, leg fatigue and RPE were seen in HIIT60% compared with MICT (table 2 and supplementary figures S3 and S4). Still, they experienced higher dyspnoea and leg fatigue compared with HCs (table 2, supplementary tables S5 and S6 and figures S3 and S4). After already 1 min of MICT, those with COPD experienced higher RPE (mean difference 2.0, 95% CI 0.2–3.2; p=0.021) and dyspnoea (1.3, 0.2–2.4) compared with HCs; p=0.032 supplementary table S6).

#### Exerkines and inflammatory markers

**FIGURE 6:**
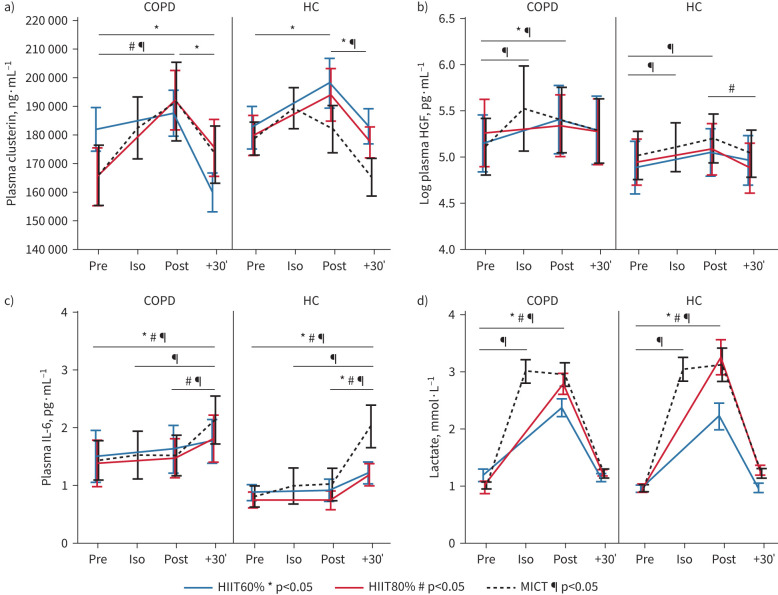
Exercise-induced change in a) plasma clusterin, b) plasma hepatocyte growth factor (HGF), c) interleukin-6 (IL-6) and d) lactate during supramaximal high-intensity interval training (HIIT) and moderate-intensity continuous training (MICT) in people with COPD and matched healthy controls (HCs). Multiple comparisons were made with Tukey's *post hoc* test after a significant main effect: *: significant difference (p<0.05) between time points for HIIT60%; ^#^: significant difference (p<0.05) between time points for HIIT80%; ^¶^: significant difference (p<0.05) between time points for MICT. HIIT60%: supramaximal HIIT at 60% of maximum mean power output for 6 s; HIIT80%: supramaximal HIIT at 80% of maximum mean power output for 6 s.

Generally, measured markers behaved similarly in those with COPD and HCs. Regarding neurotrophic factors, plasma clusterin increased after MICT (18±24%; p=0.005) and HIIT80% (22±42%; p=0.012) for people with COPD and HIIT60% in HC (10±11%; p=0.009; figure 6; supplementary tables S7 and S8 and supplementary figure S5), but with no significant difference in change between the modalities (COPD, p=0.062; HCs, p=0.224). Serum BDNF increased in MICT and HIIT80% in HCs (p<0.001), but not in any session for those with COPD (supplementary table S9 and supplementary figure S6). Lactate increased modestly and similarly in all sessions (p<0.001, figure 6 and supplementary table S10). Plasma hepatocyte growth factor (HGF) increased during all sessions. However, after adjustment for changes in plasma volume, the increase was significant only for HIIT60% and MICT in COPD and MICT for HCs (p<0.05; figure 6 and supplementary table S11).

Interleukin (IL)-6 increased from pre-exercise to 30-min post-exercise during all exercise in both groups (figure 6 and supplementary table S14). In COPD, a minor increase in IL-8 was seen during HIIT60% (supplementary table S15). No noteworthy change was seen in cathepsin B, irisin, IL-10, IL-15, TNF and *VE*GF-A during any session (supplementary tables S12–S19).

#### Harms

No serious adverse events occurred. During the cool-down phase of a HIIT80% session, one adverse event occurred: a moderate tachycardia episode in a participant with COPD on inhaler treatment. The participant was also on dual anti-arrhythmic therapy for symptomatic tachycardia due to persistent atrial fibrillation. The participant remained asymptomatic and blood pressure remained stable.

## Discussion

This study is the first to assess the feasibility and acute physiological responses of a novel watt-controlled SupraHIIT protocol compared with MICT in people with COPD. Our key findings include: 1) in COPD, SupraHIIT is feasible and enabled up to 3.5-fold increase in external exercise intensity compared with MICT, regardless of baseline exercise capacity or airflow obstruction; 2) the ventilatory demand was significantly lower in SupraHIIT despite exercising at higher intensities; 3) SupraHIIT led to clinically relevant [44] reductions in symptoms and was the preferred modality for 84% of participants; and 4) both modalities increased pBDNF, with SupraHIIT eliciting an increase despite its lower volume and producing a higher increase per min of exercise. Importantly, the pBDNF increase during SupraHIIT was independent of exercise capacity or airflow obstruction, unlike MICT, where lower capacity and more severe airflow obstruction were associated with a smaller increase in pBDNF.

### Feasibility, exercise intensity and cardiorespiratory demand

Our findings that both people with COPD and HCs exercised at a similar relative intensity (% MAP) during SupraHIIT suggest that this modality is feasible and effective, regardless of ventilatory limitation or exercise capacity. Thus, this provides novel data on individuals with COPD and further supports the feasibility of SupraHIIT in HC [26, 27].

Those with COPD tolerated SupraHIIT better than MICT, with completion rates of 100% *versus* 69%. This contrasts with Nymand *et al.* [45], who found greater adherence to a 4×4-min HIIT protocol over a 10×1-min protocol, attributing intensity as a barrier to adherence. However, our data show that even higher external intensities are manageable when the work-to-rest ratio is 6:54, providing sufficient ventilatory relief. Although previous longitudinal studies report similar attendance rates between HIIT and MICT [22], the long-term feasibility and attendance of SupraHIIT among individuals with COPD remains to be investigated.

The intermittent nature of this short-duration, low-volume SupraHIIT protocol, coupled with the lower ventilatory demand and perceived dyspnoea is a plausible reason for the high feasibility and preference of SupraHIIT [20, 23]. While the external intensity (W) is supramaximal, the cardiorespiratory intensity measured as *V′*_O_2__ and HR, reveals an intensity on the border of moderate to vigorous [10]. In COPD, peak dyspnoea during SupraHIIT was 2.0 (HIIT60%) and 0.8 (HIIT80%) points lower than MICT. However, it is important to consider that breathlessness during SupraHIIT is intermittent, with ratings being collected directly after a 6-s bout. Whereas for MICT, breathlessness is constant.

Based on the cardiorespiratory demand and perceived symptoms, we would hypothesise that SupraHIIT has greater potential for progression in both intensity and duration (*i.e.* number of intervals) than MICT, where ventilatory constraints may more readily hinder progression. This aligns with previous findings that intermittent exercise elicits lower ventilatory demand and dyspnoea in COPD [20, 23]. Also, the high mechanical loading achieved in SupraHIIT is an interesting stimulus that could benefit skeletal muscle adaptations [46, 47]. Whether or not SupraHIIT can lead to superior extrapulmonary adaptations compared with MICT remains unexplored and warrants further investigation [48].

Regarding safety, the isolated case of transient tachycardia during cool-down in SupraHIIT was likely patient-specific rather than directly attributable to the exercise modality. Given the comparable cardiopulmonary responses, characterised by similar or lower heart rates, comparable blood pressure responses and maintained oxygen saturation, SupraHIIT does not seem to pose a greater cardiovascular risk than MICT. Notably, HIIT has been shown to be safe in cardiac rehabilitation [49]. Still, the long-term safety of SupraHIIT warrants further investigation in larger, longitudinal trials, particularly in individuals with more severe COPD and cardiovascular comorbidity.

### Exercise-induced neurotrophic factors

This is the first study to measure HIIT-induced BDNF changes in COPD, showing that both SupraHIIT and MICT increased pBDNF by approximately 59% (range 30–87%; supplementary table S3), comparable with or exceeding findings in healthy and young populations. Notably, while MICT was performed on a moderate external intensity, its metabolic intensity was vigorous to near-maximal, potentially explaining the lack of between-modalities effects. Importantly, as one-third of COPD participants could not complete MICT, its intensity would require reduction in clinical settings, potentially diminishing the BDNF response [9]. In contrast, all participants with COPD completed both SupraHIIT protocols without modifications, highlighting its feasibility for inducing profound pBDNF increases in this population despite its low work volume. In fact, pBDNF increased 5–10-fold more per min of exercise at target intensity in SupraHIIT compared with MICT. Notably, all participants could complete SupraHIIT which suggests that those who completed comfortably, may tolerate a higher intensity and thereby receiving a potentially greater response.

In the general population, Dinoff *et al.* [15] found an approximate 40–60% increase in BDNF following acute exercise, with higher intensity and longer durations linked to greater responses. They suggested that a minimal duration of 30-min is needed for an increase in BDNF, and that the magnitude is attenuated with older age and lower cardiorespiratory fitness [15]. Our study demonstrates that 1 min of SupraHIIT (10×6-s intervals) within a 10-min time commitment, plus a low-intensity warm-up was sufficient to significantly increase pBDNF among elderly adults with and without COPD and a reduced cardiorespiratory fitness. This indicates an impressive BDNF response in relation to exercise duration and the total work which is half in SupraHIIT compared with MICT.

The novel findings indicating that both SupraHIIT and MICT enable a profound acute increase in pBDNF are important. Current evidence points to BDNF as a key regulator of cognitive performance and brain health [50, 51]. Exercise-induced increases in BDNF concentrations are associated with improved cognitive performance, attention and spatial memory [11, 12, 52].

The modest increase in lactate driven by increased energy demand and glycolytic flux is expected. As an important exerkine [53], lactate crosses the blood–brain-barrier (BBB) *via* monocarboxylate transporters and promotes hippocampal BDNF expression [52, 54] through the cerebral SIRT–PGC-1α–FNDC5 pathway [54], recognised as an important mechanism for brain health [14, 55]. Additionally, exercise-induced PGC-1α upregulation in skeletal muscle increases FNDC5 expression, which is cleaved and released into circulation as irisin, a myokine suggested to cross the BBB [50] and promote BDNF expression in the brain. However, irisin detection remains controversial [56] and its physiological role is still to be fully understood [57]. Thus, the absence of a significant increase in irisin in our study may be attributed to exercise protocol design or methodological reasons. Similarly, cathepsin B, another muscle-derived factor proposed to cross the BBB and stimulate BDNF production [58], remained unchanged in both exercise modalities.

Beyond our measurements, another PGC-1α-dependent mechanism involves the conversion of kynurenine to kynurenic acid, which may contribute to exercise-induced neuroprotection and BDNF modulation [59]. Furthermore, skeletal muscle produces BDNF [60] potentially promoting neurogenesis and increased hippocampal BDNF levels [59]. Other mechanisms, such as increased neuronal activity and cerebral blood flow, have been proposed [52, 59].

In sum, the increase in pBDNF may be partly driven by activation of the SIRT1–PGC-1α–FNDC5 pathway. As lactate levels remain modest in both modalities, other lactate-independent mechanisms involving upregulated PGC-1α and additional pathways, may contribute to the pBDNF elevation seen here.

Considering the high prevalence of cognitive impairments in COPD compared with non-COPD populations and its detrimental consequences, actions are needed to optimise the treatment of cognitive impairment in COPD [61, 62]. Our finding of a significant pBDNF response following SupraHIIT and MICT is promising and warrants further investigation.

In contrast to our findings, de Araujo *et al.* [63] reported decreased pBDNF following a pulmonary rehabilitation session combining walking and resistance training. They attributed this to reduced BDNF excretion and increased clearance during recovery, as well as elevated cortisol levels inhibiting BDNF. However, cortisol was not measured in their study or ours. We propose that differences in pBDNF response may also be explained by differences in exercise intensity. Their protocol utilised 30 min of treadmill walking at 60% of 6-min walking-test speed, which may have been too low to elicit an acute pBDNF increase.

The attenuated increase in HIIT80% compared with HIIT60% in the COPD group was unexpected and may be attributed to a sequence effect, as the HIIT80% session was always the third session. Early-phase effects have been observed in untrained individuals, with a more pronounced exercise-induced hormonal change early in a training phase [64]. While speculative, we have no reason to believe that SupraHIIT at a lower intensity would be beneficial over a higher one in this context [9, 15]. Of note, the reversed pattern was seen in HCs. Given the sample size, caution is warranted in drawing conclusive interpretations from this observation.

Similarly to pBDNF, we found an increase in clusterin following both SupraHIIT and MICT, without a difference between modalities. The increase in clusterin is important as it is thought to reduce neuroinflammation and to be a mediator of the effects of exercise on improving cognitive function [65, 66]. Further, the increase in HGF, suggested as a neurotrophic, pro-angiogenic, cardioprotective and anti-inflammatory factor is in line with previous research [67, 68] and potentially beneficial. Yet, research on exercise-induced change in clusterin and HGF is still in its infancy.

#### Cytokine response

The cytokine response was similar between the modalities with an increase in IL-6 30-min post-exercise, but no noteworthy change in other pro- or anti-inflammatory marker. This aligns with previous studies in COPD that have found small acute increases in inflammatory markers [69, 70]. The exercise-induced rise in IL-6 is beneficial, given its pleiotropic benefits—including anti-inflammatory actions, muscle growth, lipolysis [71, 72] and cardiac remodelling [73]. Proinflammatory cytokines IL-1β and TNF did not increase in any case, which is in agreement with the literature [74]. This underlines that the cytokine cascade induced by exercise, including supramaximal intensities in COPD, differs from that induced by infections, leading to a net long-term anti-inflammatory effect [72]. In turn, it could be an important contributor to not only reduce cognitive impairments in the COPD population [62], but also to improve extrapulmonary manifestations including cardiovascular health and function, muscle dysfunction and depression where systemic inflammation likely plays a role [2].

#### Acute responses in COPD *versus* HC

For the first time, we demonstrate that individuals with COPD can increase pBDNF levels through exercise to a similar extent as matched HCs. This is a promising and positive finding, suggesting that they may experience BDNF-mediated brain health benefits. However, factors including systemic inflammation, neuroinflammation and oxidative stress could modulate these adaptations and require further investigation.

The lower completion rate of MICT in COPD compared with HCs is not surprising. Interestingly, this is not seen in SupraHIIT where no protocol modifications were needed. This is likely attributed to the lower ventilatory demand in SupraHIIT, supporting previous findings [20, 21].

At the same % MAP, dyspnoea and leg fatigue were higher in those with COPD during all sessions. For RPE, this was however only evident in MICT. Strikingly, after just 1 min of MICT, COPD participants reported higher dyspnoea and RPE, compared with HCs. In contrast, these differences took longer to manifest in SupraHIIT, with dyspnoea becoming higher after the fifth interval (*i.e.* fifth minute) and RPE differing only at the session's end. This suggests that COPD participants tolerated, at least the initial intervals of SupraHIIT better, with delayed onset of symptoms compared with MICT.

Cardiopulmonary responses in COPD compared with HCs have been studied primarily during low- to moderate-intensity constant-load exercise [75]. To our knowledge, it has not been studied in HIIT or SupraHIIT. Here, we show that despite similar absolute *V′*_E_ levels, COPD participants exercised closer to their MVV and had higher ventilatory equivalents for O_2_ and CO_2_, also in SupraHIIT. However, as SupraHIIT imposes a lower overall ventilatory demand than MICT, these constraints did not appear to limit the feasibility. This underscores the benefit of exercise modalities that reduce ventilatory demand. As expected, HCs exercised at a higher absolute *V′*_O_2__ due to their greater workload; however, *V′*_O_2__ % peak did not differ between groups, a promising finding observed across all modalities.

### Exercise protocols and total work

We intentionally did not match the exercise protocols for total work performed but rather designed them to reflect real-world training practices. The rationale was that HIIT, SupraHIIT or sprint interval training is a time-efficient alternative to continuous, moderate-intensity training [16, 27, 76]. The aim was to keep both the intervals and the session duration short, considering the challenges posed by ventilatory constraints and dynamic hyperinflation in COPD. Subsequently, the total work performed is two-fold in MICT compared with SupraHIIT, which should be acknowledged when drawing conclusions. Despite this, SupraHIIT induced favourable responses such as increased pBDNF.

Using an iso-work design has certain advantages for comparing effects per unit of work. This would, however, artificially extend the SupraHIIT duration with multiple intervals and remove a key feature that makes the modality appealing—its lower total time commitment and lower volume. Thus, reducing ecological validity. Work-matching would also introduce methodological challenges due to individual variations in anaerobic power reserve, meaning that some individuals can sustain higher intensities above their MAP for longer than others. This variability would make it difficult to ensure a fair comparison across participants.

### Strengths and limitations

One strength of this trial is the rigorous measurement protocol. By capturing external intensity (W), cardiorespiratory demand and perceived exertion, we gained insights into work performed and how it was experienced. Additionally, our sample was well-matched for sex, age, body composition and physical activity. Although physical activity levels were not statistically different, it should be noted that HCs had a clinically relevant higher step count [77]. Another strength is that biomarker analyses were corrected for plasma volume shifts, ensuring that observed changes reflect actual differences rather than haemoconcentration [78].

One limitation is the low amount of individuals with severe and lack of individuals with very severe airflow obstruction, which limit the generalisability to those with advanced COPD. However, we found no strong indication that degree of airflow obstruction was related to the ability to perform SupraHIIT, or the increase in exercise intensity from MICT to SupraHIIT. Moreover, our findings demonstrate that SupraHIIT is feasible for older individuals with multimorbidity. Last, although the sample size was deemed sufficient to detect relevant differences in exercise intensity and pBDNF levels, it may have been insufficient to detect other differences, for example between COPD and HCs and these comparisons should be interpreted carefully.

### Conclusion

In conclusion, this study demonstrates the feasibility of short-duration SupraHIIT in achieving very high external exercise intensities, elevated pBDNF levels and the release of other potentially beneficial exerkines. Although MICT also increased pBDNF and exerkines, SupraHIIT achieved these benefits with lower volume of work, lower ventilatory demand and reduced dyspnoea, offering a more time-efficient and feasible exercise option, which also was the preferred mode of exercise. Further research should investigate the long-term feasibility and extrapulmonary benefits of SupraHIIT in this population, including individuals with more-advanced COPD.

## Data Availability

The data that support the findings of this study and statistical model outputs are available from the corresponding author upon reasonable request. Data are located in controlled access data storage at Umeå University.

## Supplementary material

**Please note:** supplementary material is not edited by the Editorial Office, and is uploaded as it has been supplied by the author. Supplementary material 01321-2024.SUPPLEMENT
