# Supplementary material for: Feasibility and acute physiological responses to supramaximal high-intensity interval training in COPD: a randomised crossover trial
Source: ERJ Open Res. 2025 Sep 22;11(5):01321-2024. doi: 10.1183/23120541.01321-2024 (PMC12451572; doi:10.1183/23120541.01321-2024)
Supplement: Supplementary file 1 [file 01321-2024.SUPPLEMENT.pdf]

Supplementary materials

Feasibility and acute physiological responses to supramaximal high-intensity interval-training in COPD: A randomised crossover trial

Authors: Johan Jakobsson, Jana De Brandt, Mattias Hedlund, Anna-Clara Rullander, Thomas Sandström, André Nyberg

Table of content

Supplementary methods **2**
Screening and recruitment 2
Eligibility criteria 2
General procedures 2
Exercise protocols 3
Measurements during exercise 4
Outcomes 6
Statistical methods 7
Supplementary results **8**
Participants. 8
Primary outcomes 8
Plasma brain-derived neurotrophic factor (Table S2-S4, Figure S2) 8
Secondary outcomes 10
Cardiorespiratory demand and exertion during exercise and at end of exercise (Table S5, Figure S3) 10
Perceived exertion during exercise (Table S6, Figure S4). 10
Exerkines and neurotrophic factors (Table S7-S13, Figure S5-S6). 17
Inflammatory markers (Table S14-S19) 22
Supplementary references **26**

Supplementary methods

Screening and recruitment

We used a convenience sampling method to select study participants. People with COPD were recruited via two pathways: i) people with COPD were referred to the research team by a COPD nurse at University Hospital of Umeå; and ii) people with COPD, that previously participated in scientific studies and consented with being contacted again for future studies, were contacted by the research team. Healthy older adults were recruited via advertisement.

Potentially eligible participants were contacted with a phone call by a physiotherapist or exercise physiologist to screen for eligibility. After giving their consent, potential participants' medical records were screened by a physician for any medical contradiction warranting exclusion. Diagnosis of COPD and pulmonary function were controlled through medical records and baseline testing, respectively. Subsequently, participants attended the facility four times within 14 days with at least 48 hours of rest between visits to allow for complete rest.

Eligibility criteria

Inclusion criteria: 1) ≥60 years of age; 2) diagnosis of COPD based on post-bronchodilator spirometry with a forced expiratory volume in one second over forced vital capacity (FEV_1/FVC) < 0.7[1] (COPD only). Exclusion criteria were: 1) neuromuscular, orthopaedic and/or any other condition that compromise participation in exercise testing; 2) unstable cardiac disease and/or the presence of a cardiac stimulator; and 3) diagnosis and treatment of lung cancer in the last five years. A pulmonologist judged eligibility based on exclusion criteria 1 and 2. Additional COPD-specific exclusion criteria were: 1) an acute exacerbation of COPD in the last six weeks. Additional exclusion criteria for healthy controls were: 1) any respiratory disease; and 2) too low or high number of steps per day that prohibited physical activity matching.

General procedures

Participants were instructed to refrain from vigorous physical activity 48 hours before each visit. In addition, they were instructed to refrain from caffeine intake and smoking six and eight hours prior each visit, respectively while continuing to adhere to their regular medication routines. To control for circadian fluctuations on performance and physiological effects, visit 2, 3 and 4 were scheduled at the same time of day (9.00 AM or 1.00 PM).

Pulmonary function tests

Pulmonary function tests were performed using Vyntus ONE system (Vyaire Medical Inc. IL, USA for forced and slow spirometry[2], static and dynamic lung volumes[3] using body plethysmography and diffusion capacity[4] following ATS/ERS guidelines. The Global Lung Function Initiative calculator (https://gli-calculator.ersnet.org/) was used to calculate percent predicted values for forced-expiratory volume in 1 second (FEV_1). Other reference equations for men[5], women[6], those above 71 years of age[7-9], were used to calculate expected values for forced spirometry measures, lung volumes and diffusion capacity for carbon monoxide based on sex, age, and height.

Cardiopulmonary exercise test

The CPET was performed on a stationary electronically-braked ergometer (Rodby RE990, Rodby Innovation AB, Vänge, Sweden) according to ERS guidelines[10]. Participants were equipped with a facemask (Vyaire Medical), to perform gas sampling and volume transduction for the measurement

of inspired and expired gas volumes and airflow variables using indirect calorimetry via a metabolic cart with breath-by-breath technology (Vyntus CPX, Vyaire, Mettawa, IL, USA), a 12-lead electrocardiography (ECG; EC Sense, Cardiolex, Solna, Sweden) and a forehead sensor for peripheral oxygen saturation (8000R, Vyaire, Mettawa, IL, USA). Gas calibration and automatic volume calibration of the metabolic cart were performed before every test. Maximal encouragement was given during the test.

The CPET was performed as follows: Participants rested for three minutes on the bike, followed by three minutes of warm-up at the lowest possible load (20 Watt) at a pedalling cadence of 50 to 70 revolutions per minute (RPM). Then, a test phase with an incremental ramp protocol at a pedalling cadence of 50 to 70 RPM, and three minutes of cool-down at the lowest wattage at a slower pedalling cadence of 30 to 50 RPM was conducted. The choice of ramp protocol (7 to 20 W/min) was guided by normative values (based on age, sex, height, disease severity (only for COPD), physical activity and estimated exercise capacity based on consultation with the participant) for expected MAP. Prediction equations for MAP% was based on Brudin formula[11], while Gläser formula[12] was used for VO_2peak% predicted.

Baseline characteristics
To characterise the participants, we obtained the following data during the first visit via intake conversation and assessment: anthropometrics including height, weight, body mass index (BMI), fat mass and fat-free mass index (FFMI) (bioelectrical impedance, Tanita BC-418MA, TANITA, United Kingdom); the impact of COPD and breathlessness using COPD assessment test (CAT) and the modified Medical Research Council dyspnea scale (mMRC); pharmacological therapy, smoking status, smoking history (pack-years), number of COPD exacerbations and hospitalisations in the previous 12 months.

Objectively measured physical activity
To match participants for physical activity (PA), it was measured using tri-axial accelerometery (DynaPort MoveMonitor, McRoberts, The Hauge, The Netherlands). This valid[13] accelerometer is worn on the lower back with a stretchable band and weighs only 55 g. Included participants with COPD wore the monitor for seven consecutive days, including two weekend days, starting the measurement when they finished the first visit. The monitor was removed during showers and water-based activities only. Days with study visits were excluded from the PA analysis, and a valid measurement required ≥4 valid days of measurements, defined as ≥8 hours of waking wear time. Potentially eligible healthy controls wore the PA monitor, also for seven consecutive days, before trial entry because too high levels of PA were a reason for exclusion.

Exercise protocols
All exercise modalities started with a five-minute warm-up and ended with a five-minute cool-down, performed at an intensity corresponding to 30% of MAP with a self-selected pedalling cadence of 60-70 RPM.

Following warm-up, the supramaximal HIIT was performed as 10×6 second intervals at 80-90 RPM, interspersed with a 54-second recovery between intervals, of which the 24 first seconds was passive, and the last 30 seconds active recovery at 30% of MAP with 60-70 RPM. The intensity during intervals was individualized and set as 60% of MPO_6 (called HIIT60%) derived from the mBCST, corresponding to ≈150% of MAP. The total duration of the session was 20 minutes. The MICT was performed at 60%

of MAP for 20 minutes with a at 60-70 RPM, according to guidelines[14] for people with COPD. The total duration for the MICT session was 30 minutes. Lastly, the fourth visit consisted of a second session of the supramaximal HIIT protocol, but the intensity was now set at 80% of MPO_6 (called HIIT80%), i.e., ≈200% of MAP. Figure S1 depicts an overview of the exercise protocol for a representative participant.

The HIIT80% session was predetermined as the last session since we first wanted an unbiased comparison between HIIT60% and MICT, not knowing the feasibility of the modality. All exercises were supervised by a physiotherapist or exercise physiologist. Standardised, moderate encouragement was given during all sessions. As we aimed to investigate the feasibility and physiological response to the exercise modalities performed as they would typically be applied in a real-world setting, the modalities are not matched for total work.

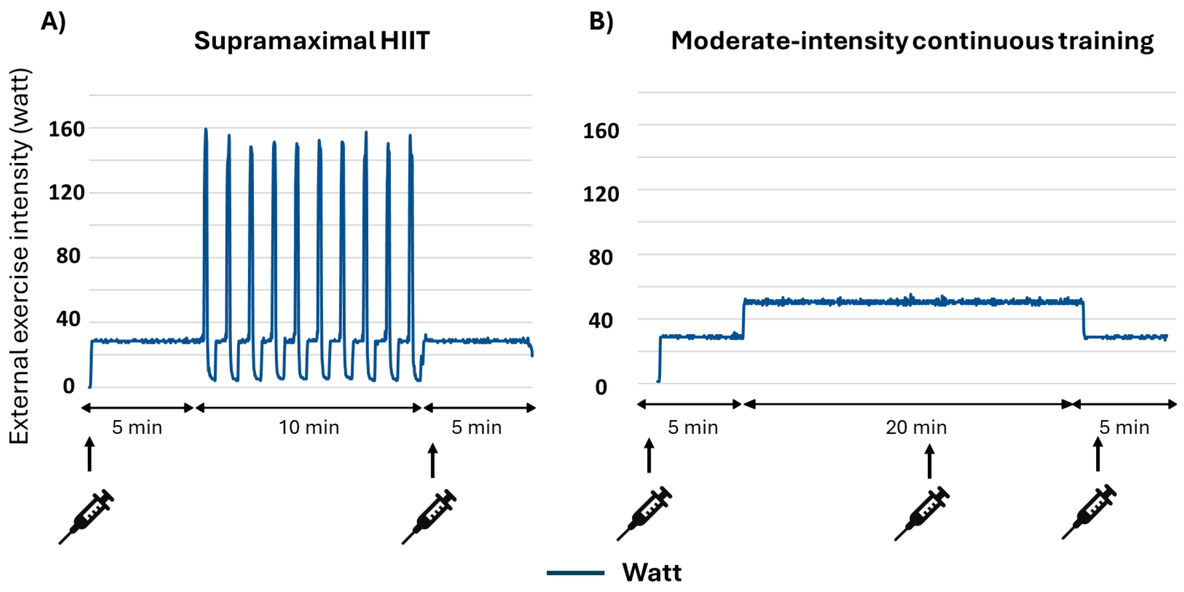

Figure S1. An overview over the exercise protocols for a representative participant. A) Depicts a supramaximal high-intensity interval-training session where intervals are performed at 80% of maximum mean power output for 6-seconds (HIIT80%). B) Depicts a moderate-intensity continuous (MICT) session, performed at 60% of max power output (MAP) during a CPET. As illustrated, venous blood samples were collected before exercise and directly after the 10 (HIIT) or 20 (MICT) main exercise block, during cool-down. In addition, an iso-time sample was collected after 10 minutes of MICT. After all sessions, a recovery blood sample was collected 30 minutes post-exercise.

Shortly before the exercise began, the participant had a short familiarization bout. For supramaximal HIIT, it consisted of three 6-second intervals at 50%, 75% and 100% of the target power for the upcoming interval session, with the same 54-second recovery in between intervals. For MICT, we kept the familiarization similar for the sake of standardisation, with the same three 6-second intervals but an intensity corresponding to the MICT.

Measurements during exercise

During all exercise sessions, we measured respiratory gas exchange with a Metamax-3B (Cortex Biophysik GmbH, Leipzig, Germany) system using the breath-by-breath method. The Metamax-3B is a valid system for indirect calorimetry[15]. Gas calibration and automatic volume calibration was performed before each test according to the manufacturer's guidelines. Oxygen uptake ($\dot{V}O_2$, mL·kg^{-1}

min^{-1}), carbon dioxide production ($\dot{V}CO_2$, L/min), and minute ventilation ($\dot{V}_E$, L·min^{-1}) were continuously measured during a 3-minute rest phase and during exercise.

Blood oxygen saturation (SpO_2, in %) was measured using a fingertip pulse oximeter (Nonin 3150, Nonin Medical, Plymouth, MN, USA). Blood pressure (BP) was measured using a valid automatic blood pressure monitor coupled with a 3-lead ECG (Tango M2, SunTech Medical, Inc., Miami, USA) and heart rate (HR) was measured with a chest strap (Polar H9, Polar Electro Oy, Kempele, Finland).

Before analysis, we removed outlier $\dot{V}O_2$ data points, defined as outside the 1.5 × interquartile range of the preceding 10 breaths. Thereafter, breath-by-breath data were conditioned to a 10-breath moving average according to recommendations[16]. For cardiorespiratory demand data, end-of-exercise was defined as the peak $\dot{V}O_2$ data point within the last 20 data-points during exercise and the first 10 datapoints during cooldown. End of exercise heart rate data, collected beat-by-beat, is on three second average rather than the 10 preceding breaths as the gas exchange parameters.

Ratings of perceived exertion (Borg RPE), dyspnea and leg fatigue (Borg CR-10) were obtained at rest, multiple times during, and after exercise: Ratings was obtained at the end of the 3-minute rest phase, at the end of the 5-minute warm-up, after minute (MICT) or interval (supramaximal HIIT) 1, 5, 10, minute 15 and 20 (MICT only), and at the end of the 5-minute cool-down. During supramaximal HIIT, ratings were collected immediately after the 6-second interval. Lastly, 30 minutes after each exercise session, session RPE[17] was collected. In addition, after their second exercise session, exercise modality preference was obtained by asking the question: *"Assuming that MICT and supramaximal HIIT would be equally effective in improving your health and physical capacity, which one would you prefer to perform?"*.

Blood analyses and materials

Blood samples was collected in conjunction with exercise sessions. Following a 15-minute seated rest, venous blood was collected by a registered nurse prior to exercise (Pre), immediately after exercise (Post), and 30 minutes after exercise (+30 min). In addition, an iso-time sample (Iso) was collected after 10-minutes of MICT. Blood was collected into K_2EDTA and serum separator tubes (Becton Dickinson, Franklin Lakes, NJ, USA). Whole blood lactate concentration (Biosen C-Line, EKF Diagnostics, Cardiff, UK), haemoglobin (Hb) concentration (Hb 201+, HemoCue, Ängelholm, Sweden) and haematocrit (Hct) (Haematokrit 200, Andreas Hettich GmbH, Tuttligen, Germany) were determined immediately. Then, tubes were put in room temperature for 30 minutes, prior centrifugation for 15 minutes at 1500g. Serum and plasma aliquots were put in -80°C within 60 minutes of sampling.

Circulating levels of plasma BDNF, HGF, IL-7, IL-13 and IL-15 (Luminex Discovery Assay, Cat. No. LXSAHM-05, R&D Systems, Minneapolis, MN, USA), serum BDNF (Luminex Discovery Assay, Cat. No. XSAHM-01, R&D), plasma IL-1β, IL-4, IL-6, IL-8, IL-10, TNF-α and VEGF-A (Luminex High Sensitivity Cytokine Panel A, Cat. No. FCSTM09-07, R&D), plasma clusterin (Luminex Performance Panel, Cat. No. LHK2937 & LHK000, R&D) was assessed using single- and multiplex fluorescent bead-based immunoassays (Luminex Corporation, Austin, TX, USA) using commercially available kits with a Bio-Rad Bioplex 200 (Bio-rad Laboratories Inc, CA, USA). Plasma cathepsin B (Cat. No. ab119584, Abcam, Cambridge, UK) and serum irisin (Cat. No. EK-067-29, Phoenix Europe GmbH, Karlsruhe, Germany) was assessed with ELISA, using commercially available kits following manufacturer procedures using

a Synergy HT plate reader (Agilent Technologies Inc. CA, USA). A blinded technician performed all analyses in triplicates.

Allocation of participant's samples to 96-plates was randomised. Participants had all their ten samples on the same plate. Pilot kits were performed to determine dilutions for each kit. All standards, blanks, quality control samples and samples were analysed in duplicate. Analytes with a high level of samples below the level of detection ([BLD], i.e. >50%, Table S1) were not analysed. For analyses with a low number of samples BLD (1-10%), missing data was handled by the linear mixed models. Standard curve fitting was performed using manufacturer guidelines using GraphPad Prism 10.0

Notably, blood samples at iso, post and +30 min was adjusted for exercise-induced changes in plasma volume following the method by Dill and Costill[18]. In turn, this attenuated the exercise-induced increase in analytes by approximately 5%-10% by adjusting for change in plasma volume, not often done in other studies.

Table S1. Analytes and kits used for analyses in the study.

Analyte	Method	Catalog No.	Standard curve [pg/ml]	Sensitivity [pg/ml]	Dilution factor	Mean CV (%)	% <LOD
**BDNF (serum)**	Luminex Serum	LXSAHM-01	20.6 - 5000	0.320	1:50	3.5	0
**BDNF (plasma)**	Luminex	LXSAHM-06	20.6 - 5000	0.320	1:2	3.2	0
**HGF**			16.5 - 4000	1.0	1:2	4.4	0
**IL-7**			5.14 - 1250	0.410	1:2	NA	55
**IL-13**			453 - 110000	36.6	1:2	NA	62
**IL-15**			6.3 - 1550	1.01	1:2	7.8	10
**IL-1b**	Luminex	FCSTM09-7	0.34 - 1.400	0.18	1:2	NA	54
**IL-4**			7.62 - 7.800	2.54	1:2	NA	68
**IL-6**			0.854 - 3.500	0.31	1:2	3.6	4
**IL-8**			0.879 - 3.600	0.07	1:2	2.0	0
**IL-10**			0.244 - 1.000	0.24	1:2	4.6	4
**TNF-α**			0.757 - 3.100	0.54	1:2	4.2	0
**VEGF**			2.25 - 2.300	1.35	1:2	4.5	1
**Clusterin**	Luminex	LHK2937 + LHK000	664 - 483 800	64.3	1:4000	4.6	0
**Irisin**	ELISA	EK-067-29	1290 - 275000	1290	1:1	7.2	0
**Cathepsin B**	ELISA	ab119584	156 - 10000	5	1:1	7.0	0

Analytes, methods and analytic kits used in the study and their specifications.
BDNF: brain-derived neurotrophic factor; HGF: hepatocyte growth factor; IL: Interleukin; TNF-α: tumor-necros factor-alpha; VEGF: vascular endothelial growth factor; LOD: limit of detection.

Outcomes

Primary outcomes were external exercise intensity (watt, also expressed as %MAP and %MICT) and change in circulating pBDNF (pq/mL, also expressed as %-change from baseline and change per minute of exercise).

Secondary outcomes included cardiorespiratory demand during exercise and at end-of-exercise, measured using gas exchange parameters ($\dot{V}O_2$ [mL·kg^{-1} min^{-1}, L·min^{-1} and %max], $\dot{V}CO_2$ [L·min^{-1}]), ventilation ($\dot{V}_E$ [L·min^{-1}]) and its derived and related variables (Respiratory exchange ratio [RER], $\dot{V}E/\dot{V}O_2$, $\dot{V}_E/\dot{V}CO_2$, $\dot{V}_E$/MVV [%MVV], breathing frequency [min^{-1}] and V_T [L]). Also, during exercise, we measured heart rate [HR, expressed as bpm and %peak], systolic BP [mmHg], diastolic BP [mmHg] and mean arterial pressure [mmHg] and peripheral oxygen saturation (SpO_2 [%]). Also, perceived exertion (Borg RPE, 6-20 pts), dyspnea and leg fatigue (Borg CR-10, 0-10 pts), session RPE (0-10 pts) and exercise preference were obtained. Further, we examined change in circulating concentration of serum BDNF (sBDNF), serum irisin and in plasma; cathepsin B, clusterin, VEGF-A, IL-1β, IL-4, IL-6, IL-10, IL-13, IL-15, TNF-α and HGF (in pg/mL or ng/mL). Feasibility was determined by exercise fidelity defined as exercise sessions needing modifications; adherence to prescribed cadence, intensity, and duration; session completion; symptoms including RPE, dyspnea, and leg fatigue. Occurrence and severity of adverse events were assessed as safety outcomes.

Statistical methods

Continuous variables were reported as mean (SD) for normally distributed data, as determined by Q-Q plots and histograms. Non-normally distributed data was presented as median (interquartile range).

Differences in external exercise intensity, cardiorespiratory outcomes, symptoms, and exerkines concentrations, were assessed using one-way random effects repeated measures analysis of variance (RM-ANOVA) with participants as random effects. Models were fitted using restricted maximum likelihood (REML). To investigate within-group changes, models were fitted separately per group (COPD and HC) with time as fixed effects. To compare between-group differences, i.e. change in BDNF from pre- to post-exercise, group was used as fixed effect. Tukey's post hoc analysis was used for multiple comparisons following significant main effects. Log transformations were applied as needed to meet assumptions for linear-mixed models. Specifically, for plasma BDNF, IL-6, IL-8, IL-10, IL-15, HGF, TNF-alpha and VEGF-A, log-transformation was performed, yielding normal distribution and normal distributed residuals and better fit of the models. Non-transformed data are used for descriptive figures, if not mentioned otherwise.

Pairwise comparisons at baseline and different time points during exercise were analysed with independent t-tests, Welch's test, Mann-Whitney U test, or Fischer's exact test as appropriate. Quade's test with daylight time as covariate was used for physical activity analysis. We used Pearson and Spearman correlations for exploratory analyses investigating the associations of baseline characteristics to selected outcomes. The strength of the correlation coefficients will be categorized as, for Pearson r: low (0–0.25), moderate (> 0.25–0.50), strong (> 0.50–0.75) and very strong (> 0.75); For Spearmans Rho: trivial (<0.1), low (0.1–0.3), moderate (0.3–0.5), strong (0.5–0.7), very strong (0.7–0.9), and extremely strong (>0.9-1.0)[19]. Statistical analyses were conducted using JMP Pro version 17.2 with a two-sided alpha level of 0.05.

Analytes IL-1β, IL-4, IL-7 and IL-13 were not analysed due to 50% of data points being below the limit of detection (Table S1).

Supplementary results

Participants.

The primary reason for exclusion was cardiovascular abnormalities detected during the CPET requiring further investigation (Figure 1). Table 1 shows that the COPD group, while similar in age and body composition to the HC group, had a reduced lung and cardiorespiratory function. The COPD group had a mild to severe disease, more current and ex-smokers, more prescribed medications, and more comorbidities compared to HCs.

Primary outcomes

Plasma brain-derived neurotrophic factor (Table S2-S4, Figure S2)

Circulating pBDNF levels significantly increased during both supramaximal HIIT and MICT sessions in both groups (Table S2). The change from Pre to Iso or Post were not significantly different between sessions within either group (COPD: p=0.346; HC p=0.299). Additionally, no significant between-group differences in pBDNF changes were observed across any session (HIIT60%: p=0.401; MICT-Iso: p=0.919; MICT: p=0.637; HIIT80%: p=0.378). Pairwise comparisons showed no between-group difference in pBDNF at any timepoint.

Table S2. Plasma concentration of brain-derived neurotrophic factor, pg/mL

		COPD		HC		P-Time	
		**N**	**Mean (SD)**	**N**	**Mean (SD)**	**COPD**	**HC**
**HIIT60%**	**Pre**	16	3541 (2083)	15	2959 (1988)	**0.001**	**0.017**
	**Post**	15	5163 (2945)[a]	15	4076 (2313)[a]		
	**30 min**	15	3430 (1637)[b]	15	3408 (2330)		
**MICT**	**Pre**	16	3356 (1891)	16	3287 (1975)	**0.004**	**<0.001**
	**Iso**	12	4498 (2158)[a]	16	4602 (2437)[a]		
	**Post**	16	4933 (2954)[a]	16	5271 (2694)[a]		
	**30 min**	16	4233 (3138)	16	3404 (1975)[bc]		
**HIIT80%**	**Pre**	15	3929 (1947)	15	3030 (1388)	**0.018**	**<0.001**
	**Post**	15	4675 (2443)[a]	15	4358 (2972)[a]		
	**30 min**	15	3870 (1941)[b]	14	2814 (1356)[b]		

Supramaximal high-intensity interval-training at 60% (HIIT60%) and 80% (HIIT80%) are performed at 60% and 80%, respectively of maximum mean power output for six seconds. Moderate intensity continuous training (MICT) is performed at 60% of maximal aerobic power. COPD: chronic obstructive pulmonary disease; HC: healthy control

Tukey's post hoc comparisons were made after a significant main effect: [a] = significantly different from Pre (p<0.05), [b] = significantly different from Post (p<0.05), [c] = significantly different from Iso (P<0.05)

To illustrate the relative increase in pBDNF, the mean percent change-from-baseline in pBDNF is shown in Table S3 and Figure S2a, which ranged from 30% to 87% in COPD, and 37% to 57% in HC. As descriptively shown in Figure S2b, the general exercise-induced response in relative pBDNF increase was similar between those with COPD and HC.

Table S3. Percent change in plasma brain-derived neurotrophic factor (BDNF) from baseline

		COPD		HC	
		**N**	**Mean (SD)**	**N**	**Mean (SD)**
**HIIT60%**	**Post**	15	64 (67)	15	57 (59)
	**30 min**	15	17 (62)	15	39 (92)
**MICT**	**Iso**	12	78 (97)	16	56 (61)
	**Post**	16	87 (117)	16	77 (82)
	**30 min**	16	46 (84)	16	9 (28)
**HIIT80%**	**Post**	15	30 (45)	15	37 (58)
	**30 min**	15	4 (29)	14	-7 (31)

Supramaximal high-intensity interval-training at 60% (HIIT60%) and 80% (HIIT80%) are performed at 60% and 80%, respectively of maximum mean power output for six seconds. Moderate intensity continuous training (MICT) is performed at 60% of maximal aerobic power. COPD: chronic obstructive pulmonary disease; HC: healthy control

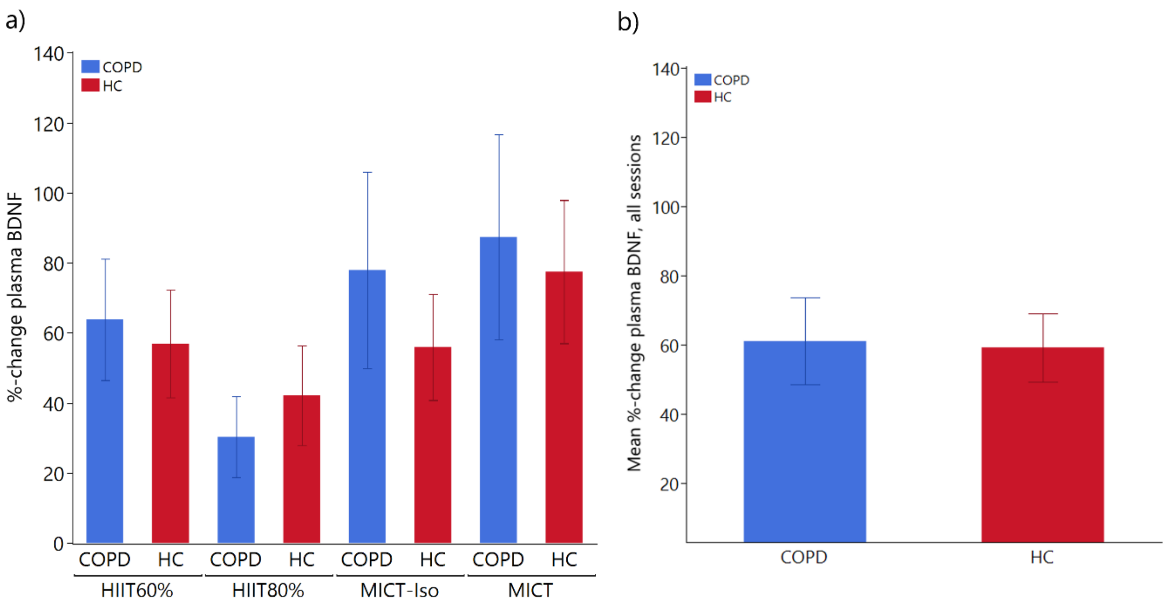

Figure S1. Exercise induced change in plasma brain-derived neurotrophic factor (BDNF) during supramaximal high-intensity interval training (HIIT) and moderate-intensity continuous training (MICT) in people with chronic obstructive pulmonary disease (COPD) and matched healthy controls (HC). Data is shown as mean±SE. Panel **a)** shows the relative change (%) from baseline to post-exercise and MICT-Isotime. Panel **b)** shows the mean change from baseline to post-exercise in COPD and HC, showing that the relative increase in similar between groups.

When normalised per minute of exercise at target power, supramaximal HIIT produced a substantially greater increase than MICT. The duration-adjusted increase in pBDNF was significantly higher during HIIT60% compared to MICT (p=0.002) and MICT-Iso (p=0.005) in people with COPD, while it was so for HIIT80% in HCs (p=0.016 for MICT, p=0.020 for MICT-Iso, Figure 4c, Table S4).

Table S4. Change in plasma brain-derived neurotrophic factor (BDNF) from baseline to immediately after exercise or MICT-Isotime, pg/mL × min^{-1}

	COPD		HC	
	**N**	**Mean (SD)**	**N**	**Mean (SD)**
**HIIT60%**	15	1575 (1793)[ab]	15	1116 (1058)
**MICT-Iso**	12	125 (229)	16	132 (134)
**MICT**	16	69 (162)	16	99 (86)
**HIIT80%**	15	746 (1598)	15	1328 (1933)[ab]

Data is shown as mean (SD). Supramaximal high-intensity interval-training at 60% (HIIT60%) and 80% (HIIT80%) are performed at 60% and 80%, respectively of maximum mean power output for six seconds. Moderate intensity continuous training (MICT) is performed at 60% of maximal aerobic power. COPD: chronic obstructive pulmonary disease; HC: healthy control.

[a] significantly different from MICT ($p<0.05$), [b] significantly different from MICT-Iso ($p<0.05$)

Secondary outcomes

Cardiorespiratory demand and exertion during exercise and at end of exercise (Table S5, Figure S3)

Table S5 (below) shows the cardiorespiratory demand, perceived exertion, dyspnea and leg fatigue at the end of each exercise sessions, as well as iso-time in MICT, i.e. after 10 minutes. As seen, the cardiometabolic ($\dot{V}O_2$, HR) and ventilatory demand ($\dot{V}E$, $\dot{V}E$/MVV, BF) was in general higher during the end of MICT compared to both sessions of supramaximal HIIT. Those with COPD showed a lower ventilatory efficiency ($\dot{V}E/\dot{V}O_2$ and $\dot{V}E/\dot{V}CO_2$ $p<0.05$ for all, Table S5) at end-of-exercise in all sessions compared to HC. Figure S3 depicts %$\dot{V}O_2$peak, %HRpeak, RER and dyspnea during exercise (data averaged from after warm-up until, but excluding, cool-down).

Perceived exertion during exercise (Table S6, Figure S4).

Table S6 (below) shows the perceived dyspnea, leg fatigue and RPE at before, multiple times during, and after each exercise as well as session RPE. As seen, MICT generally induced higher levels of dyspnea, leg fatigue and RPE. Also, especially during MICT but also supramaximal HIIT, those with COPD experienced higher levels of dyspnea, leg fatigue and RPE depicted in Figure S4.

Table S5. Cardiorespiratory demand, perceived exertion, dyspnea and leg fatigue at end-of-exercise and iso-time.

	**HIIT60%**			**HIIT80%**			**MICT-ISO**			**MICT**		
	**COPD**	**HC**	**P-Group**	**COPD**	**HC**	**P-Group**	**COPD**	**HC**	**P-Group**	**COPD**	**HC**	**P-Group**
$\dot{V}O_2$ ($L \cdot min^{-1}$)	1.2 (0.2)[abc]	1.5 (0.2)[abc]	**<0.001**	1.3 (0.3)[a]	1.6 (0.3)[a]	**0.003**	1.4 (0.3)	1.6 (0.3)[a]	**0.029**	1.4 (0.3)	1.8 (0.3)	**0.004**
$\dot{V}O_2$ ($mL \cdot kg^{-1} \cdot min^{-1}$)	16.5 (3.2)[abc]	20.2 (2.2)[ab]	**0.001**	18.3 (3.8)[a]	21.7 (3.7)[a]	**0.017**	19.0 (3.4)[a]	22.1 (3.3)[a]	**0.018**	20.4 (4.1)	24.0 (3.7)	**0.012**
$\dot{V}O_2$ (%max)	77 (14)[abc]	80 (7)[abc]	0.586	85 (13)[a]	86 (15) [a]	0.907	84 (9)[a]	88 (10)[a]	0.387	95 (14)	95 (12)	0.969
HR (bpm)	106 (14)[ac]	115 (12)[abc]	0.068	110 (16)[a]	125 (16)[a]	**0.012**	113 (16)[a]	130 (13)[a]	**0.005**	119 (16)	138 (15)	**0.002**
HR (%peak)	77 (8)[ac]	76 (5)[abc]	0.856	80 (7)[a]	83 (7)[ac]	0.207	82 (7)[a]	87 (5)[a]	**0.043**	86 (8)	92 (6)	0.050
RER	0.91 (0.03)	0.90 (0.03)[b]	0.758	0.93 (0.05)	0.95 (0.04)[ac]	0.193	0.93 (0.04)	0.92 (0.03)[b]	0.455	0.93 (0.05)	0.92 (0.02)	0.382
$\dot{V}E$ (L)	44.8 (8.9)[a]	43.6 (9.3)[abc]	0.713	50.1 (11.8)	53.8 (13)	0.407	49.4 (14.6)	48.7 (10.4)[a]	0.890	54 (16.3)	56.4 (12)	0.641
$\dot{V}E$/MVV (%)	0.65 (0.16)[a]	0.44 (0.11)[ab]	**<0.001**	0.73 (0.18)	0.53 (0.1)	**<0.001**	0.71 (0.19)	0.49 (0.08)[a]	**<0.001**	0.80 (0.2)	0.58 (0.13)	**0.018**
$\dot{V}E/\dot{V}O_2$	34.2 (5.4)	26.5 (3.8)[ab]	**<0.001**	35.1 (5.4)	31.0 (4.9)[c]	**0.030**	32.3 (4.7)	27.3 (4)[a]	**0.005**	34.3 (6.1)	29.4 (4.7)	**0.027**
$\dot{V}E/\dot{V}CO_2$	37.8 (6.0)	29.5 (4.0)[ab]	**<0.001**	37.9 (6.4)	32.5 (4.8)[c]	**0.012**	34.7 (5.2)	29.7 (4.2)[a]	**0.007**	36.9 (6.7)	32.0 (5.1)	**<0.001**
BF (min^{-1})	31.5 (5.6)	26.3 (4.1)[ab]	**0.008**	32.4 (4.5)	30.2 (6.4)	0.271	29.1 (5.3)[a]	28.5 (4)	0.711	34.2 (6.4)	31.2 (5.6)	0.175
$\dot{V}CO_2$ ($L \cdot min^{-1}$)	1.07 (0.2)[abc]	1.35 (0.21)[abc]	**<0.001**	1.2 (0.25)[a]	1.53 (0.3)	**0.003**	1.29 (0.3)	1.52 (0.31)	0.059	1.33 (0.31)	1.63 (0.31)	**0.081**
V_T (L)	1.5 (0.4)[c]	1.7 (0.3)[ab]	0.112	1.6 (0.4)	1.8 (0.4)	0.127	1.7 (0.4)	1.7 (0.3)	0.816	1.6 (0.4)	1.8 (0.3)	0.081
**Cardiorespiratory parameters**												
SpO_2 (%)	95 (2)	95 (1)[c]	0.656	94 (2)	94 (2)	0.864	93 (3)	94 (1)	0.609	93 (2)	94 (1)	0.169
SBP (mmHg)	157 (28)[a]	153 (28)[a]	0.734	150 (37)[a]	151 (28)[a]	0.960	-	-	-	178 (36)	181 (24)	0.753
DBP (mmHg)	77 (14)	73 (9)	0.328	72 (12)	71 (14)	0.766	-	-	-	77 (13)	69 (12)	0.075
Mean AP (mmHg)	104 (16)	100 (10)	0.401	98 (18)[a]	98 (16)[a]	0.905	-	-	-	111 (16)	107 (13)	0.406

**Perceived exertion and symptoms at end-of-exercise and MICT-Isotime**												
Borg RPE (6-20)	14.8 (2.4)[a]	13.7 (2.5)[a]	0.236	15.8 (1.9)	14.2 (2.3)	**0.034**	15.5 (2.7)	13.3 (2.1)[a]	**0.016**	17.0 (2.1)	15.1 (2.4)	**0.025**
Dyspnea (0-10)	5.1 (2.3)[ab]	3.6 (1.5)[a]	**0.036**	6.3 (1.9)	4.3 (1.8)	**0.004**	5.8 (2.0)	3.8 (1.7)[a]	**0.009**	7.1 (1.9)	5.1 (2.3)	**0.009**
Leg fatigue (0-10)	4.7 (1.9)[ab]	3.3 (1.7)[ab]	**0.027**	6.1 (1.7)	4.3 (2.2)	**0.012**	5.5 (2.3)	3.9 (1.8)[a]	**0.035**	7.0 (1.7)	5.0 (2.5)	**0.013**

Cardiorespiratory parameters, blood pressure, perceived exertion and symptoms end-of-exercise and at MICT-isotime, i.e. at 10 minutes of MICT. Data is shown as mean (SD) if not otherwise mentioned. Indirect calorimetry data, blood pressure and perceived exertion/symptoms are averages over the whole exercise session (10 minutes of supramaximal HIIT, up to 20 minutes of MICT), excluding warm-up and cool down. n=16 for all except HC HIIT60% where n=14 for VO2/VCO2-related variables and n=15 for VE-related variables due to technical issues. Supramaximal HIIT at 60% (HIIT60%) and 80% (HIIT80%) are performed at 60% and 80%, respectively of maximum mean power output for six seconds. MICT is performed at 60% of max aerobic power. COPD: chronic obstructive pulmonary disease, HC: healthy control; W = watt; MAP: Max aerobic power; V'O2: oxygen consumption; V'CO2: carbon dioxide production; HR = heart rate; RER: respiratory exchange rate; V'E: minute ventilation; MVV: maximal voluntary ventilation; BF: breathing frequency; VT: tidal volume; SpO2: peripheral oxygen saturation; SBP: systolic blood pressure; DBP: diastolic blood pressure; AP; arterial pressure; RPE: rating of perceived exertion. Tukey's post hoc comparisons were made after a significant main effect: [a]: significantly different from MICT ($p<0.05$), [b]: significantly different from HIIT80 [c]: significant different from MICT-Iso ($p<0.05$). P-group indicates p-value for pairwise comparison with independent-t test or Mann-Whitney U test.

Table S6. Perceived exertion, dyspnea and leg fatigue during exercise.

	HIIT60%			HIIT80%			MICT			P-Session	
	COPD	HC	P-Group	COPD	HC	P-Group	COPD	HC	P-Group	COPD	HC
**RPE at**											
Rest	8.0 (1.7)	7.4 (1.7)	0.299	8.3 (2.0)	7.1 (1.4)	0.482	8.1 (1.9)	6.9 (1.5)	0.076	0.621	0.305
End of warm-up	10.9 (2.6)	9.4 (2.3)	0.111	10.7 (2.7)	9.3 (2.7)	0.153	10.9 (3.1)	9.0 (1.9)	**0.046**	0.949	0.605
1st interval/min	12.3 (3.1)	11.4 (2.7)[a]	0.397	12.4 (2.8)	12.1 (2.8)[a]	0.751	11.9 (3.0)	9.9 (1.9)	**0.032**	0.777	**<0.001**
5th interval/min	13.8 (2.4)	12.4 (2.8)	0.164	14.7 (2.2)	13.5 (2.8)	0.116	14.6 (2.5)	12.5 (2.4)	**0.022**	0.090	0.128
10th interval/min	14.8 (2.4)	13.7 (2.5)	0.233	15.8 (1.9)	14.2 (2.3)	**0.034**	15.5 (2.7)	13.3 (2.1)	**0.016**	0.125	0.126
15 minutes	-	-	-	-	-	-	16.5 (2.8)	14.8 (2.1)	0.068	-	-
20 minutes	-	-	-	-	-	-	16.3 (2.1)	15.1 (2.4)	0.207	-	-
At stop*	14.8 (2.4)[a]	13.7 (2.5)	0.233	15.8 (1.9)[)a]	14.2 (2.3)	**0.034**	17.0 (2.1)	15.1 (2.4)	0.207	**0.001**	**0.022**
End of cool-down	12.9 (2.7)	11.2 (2.6)[a]	0.070	13.1 (2.9)	11.1 (2.6)[a]	0.055	14.1 (1.9)	13.5 (2.9)	0.524	0.090	**<0.001**
Session RPE	3.9 (1.6)[a]	3.2 (1.3)[a]	0.149	4.6 (1.8)	3.6 (1.4)[a]	0.117	5.3 (1.9)	4.9 (2.1)	0.595	**0.008**	**<0.001**
**Dyspnea at**											
Rest	1.2 (0.9)	0.7 (0.8)	0.086	1.2 (1.2)	0.7 (0.9)	0.134	1.2 (1.2)	0.6 (0.7)	0.083	1.000	0.621
End of warm-up	2.7 (1.4)	1.8 (1.2)	0.070	2.4 (1.3)	1.6 (1.2)	0.083	2.8 (1.5)	1.6 (1.0)	**0.015**	0.625	0.826
1st interval/min	3.3 (1.7)	2.4 (1.5)	0.116	3.5 (1.5)	2.9 (1.3)a	0.226	3.2 (1.9)	1.9 (1.0)	**0.021**	0.771	**<0.001**
5th interval/min	4.5 (2.0)	3.1 (1.8)	**0.039**	5.3 (1.6)	3.5 (1.1)	**0.001**	4.8 (2.0)	3.3 (0.9)	**0.008**	0.278	0.395
10th interval/min	5.1 (2.3)[b]	3.6 (1.5)	**0.036**	6.3 (1.9)	4.3 (1.8)	**0.004**	5.8 (2.0)	3.8 (1.7)	**0.009**	**0.024**	0.102
15 minutes	-	-	-	-	-	-	6.2 (2.1)	4.3 (1.6)	**0.009**	-	-
20 minutes	-	-	-	-	-	-	6.4 (1.6)	5.1 (2.3)	0.110	-	-
At stop*	5.1 (2.3)[ab]	3.6 (1.5)[a]	**0.039**	6.3 (1.9)	4.3 (1.8)	**0.004**	7.1 (1.9)	5.1 (2.3)	**0.009**	**<0.001**	**0.003**
End of cool-down	3.8 (1.8)	2.1 (1.1)	**0.036**	4.0 (1.9)	2.5 (1.7)	**0.030**	5.1 (2.3)	3.3 (2.4)	0.138	0.165	0.052

Table S6 continued. Perceived exertion, dyspnea and leg fatigue during exercise.

	HIIT60%			HIIT80%			MICT			P-Session	
	**COPD**	**HC**	**P**	**COPD**	**HC**	**P**	**COPD**	**HC**	**P**	**COPD**	**HC**
**Leg fatigue at**											
Rest	1.0 (0.8)	0.4 (0.6)	0.285	1.2 (1.1)	0.5 (0.6)	0.403	1.0 (1.2)	0.5 (0.7)	0.180	0.629	0.783
End of warm-up	2.5 (1.3)	1.8 (1.3)	0.150	2.3 (1.2)	1.6 (1.2)	0.120	2.5 (1.5)	1.7 (1.0)	0.070	0.695	0.893
1st interval/min	3.4 (1.9)	2.6 (1.4)	0.163	3.6 (1.7)	2.7 (1.4)	0.102	3.2 (1.8)	2.2 (1.0)	0.058	0.697	0.069
5th interval/min	4.3 (1.7)	3.0 (1.6)	**0.043**	5.1 (1.8)	3.3 (1.9)	**0.012**	4.8 (2.1)	3.1 (1.1)	**0.008**	0.178	0.502
10th interval/min	4.7 (1.9)[b]	3.3 (1.7)[b]	**0.027**	6.1 (1.7)	4.3 (2.2)	**0.012**	5.5 (2.3)	3.9 (1.8)	**0.035**	**0.012**	**0.024**
15 minutes	-	-	-	-	-	-	6.4 (2.0)	4.4 (2.0)	**0.011**	-	-
20 minutes	-	-	-	-	-	-	6.8 (1.9)	5.0 (2.5)	0.051	-	-
At stop*	4.7 (1.9)[ab]	3.3 (1.7)[a]	**0.027**	6.1 (1.7)	4.3 (2.2)	**0.012**	6.8 (1.6)	5.0 (2.5)	**0.022**	**<0.001**	**<0.001**
End of cool-down	3.8 (2.9)[a]	2.4 (1.5)[a]	**0.033**	4.3 (2.0)[a]	2.5 (1.8)[a]	**0.014**	5.0 (2.1)	4.0 (2.7)	0.270	**0.001**	**0.001**

Data is mean (SD) *At stop is equivalent to post-10th interval during HIIT for all. In MICT, due to exhaustion prior to 20 minutes in the COPD group. Also, "at stop" represents ratings taken immediately after exercise termination, prior to cool-down. . Supramaximal HIIT at 60% (HIIT60%) and 80% (HIIT80%) are performed at 60% and 80%, respectively of maximum mean power output for six seconds. MICT is performed at 60% of max aerobic power. COPD: chronic obstructive pulmonary disease, HC: healthy control. RPE = Rating of perceived exertion (Borg 6-20). N = 16 for all, except for COPD at MICT 15 min: N = 13 and COPD at MICT 20 min, N = 11. N = 16 for all, except for MICT 15 min, N COPD = 13, MICT 20 min, N COPD = 11. Tukey's post hoc comparisons were made after a significant main effect: [a]: significantly different from MICT ($p<0.05$), [b]: significantly different from HIIT80%. P-group indicates p-value for pairwise comparison with independent-t test or Mann-Whitney U test.

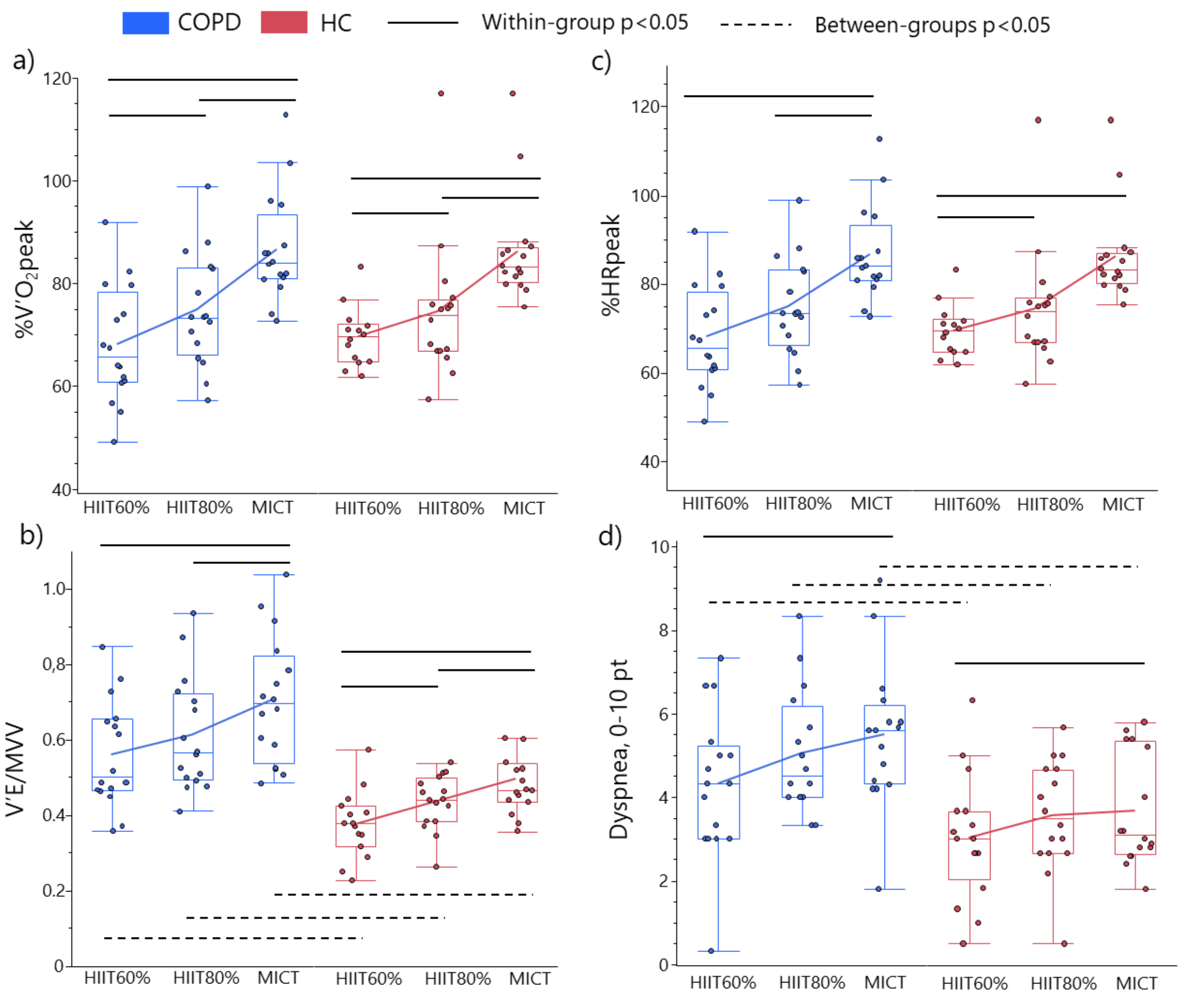

Figure S3. Boxplots depicting selected cardiorespiratory parameters during supramaximal HIIT and MICT. Indirect calorimetry data (panel **a)** $\%VO_2$peak, **b)** V'E/MVV, **c)** %HRpeak) are averaged over the whole exercise session (10 minutes of supramaximal HIIT, up to 20 minutes of MICT), excluding warm-up and cool down and dyspnea (panel **d)**) is the mean of 3-5 ratings collects during exercise. The cardiorespiratory demand (panel a-c) tended to be higher in MICT than supramaximal HIIT in both groups. While those with COPD exercised at a similar relative cardiometabolic intensity (panel a and c), they were closer to their ventilatory limit as seen in the V'E/MVV ratio in panel b). They also experienced higher levels of dyspnea compared to HC in all sessions (panel d).

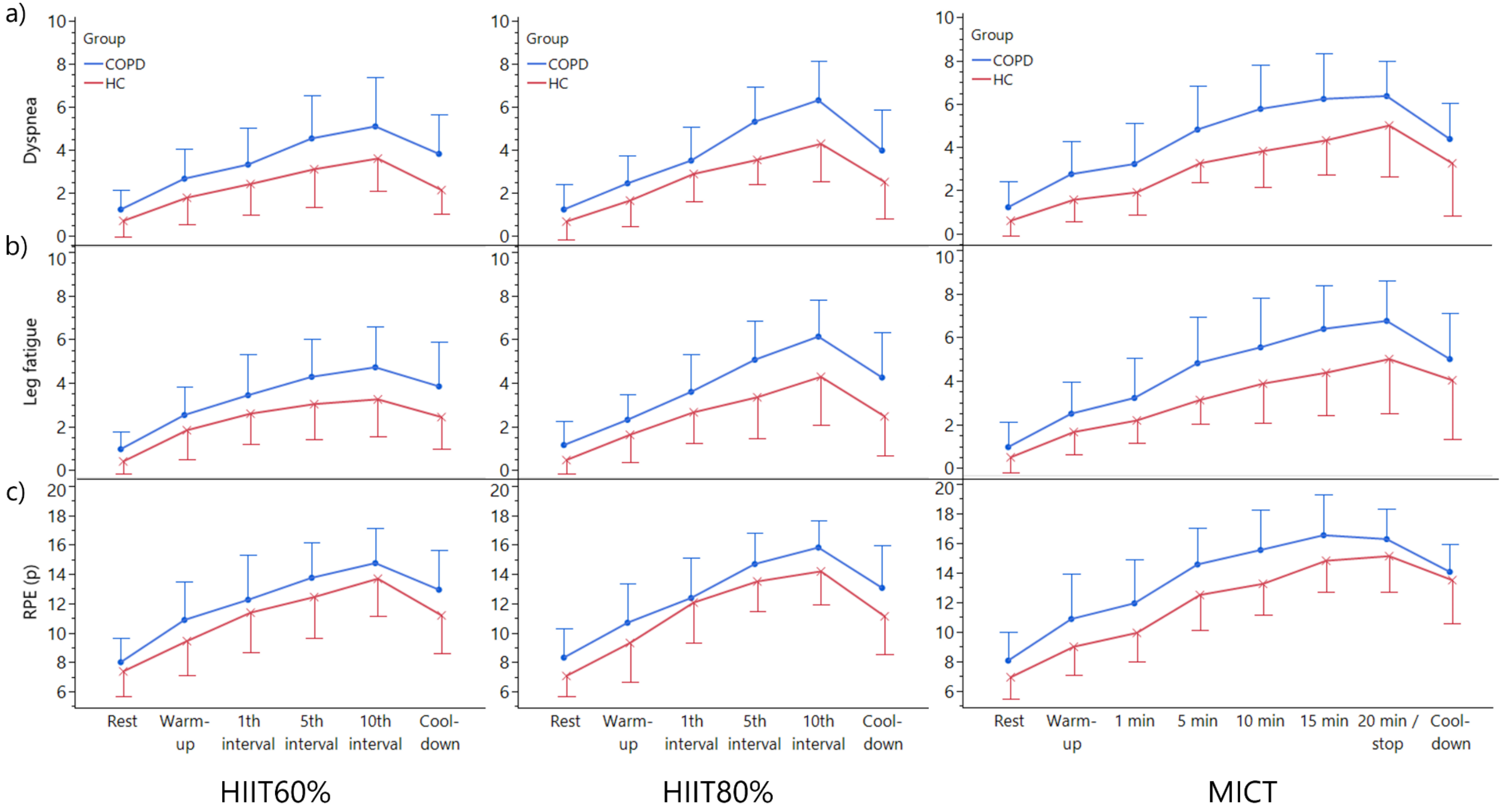

Figure S4. Descriptive plots for **a)** Dyspnea, **b)** Leg fatigue and **c)** rating of perceived exertion (RPE) during supramaximal HIIT at 60% (left panels) and 80% (middle panels) of maximum mean power output for 6 seconds, and during moderate-intensity continuous training (MICT, right panels) in people with chronic obstructive pulmonary disease (COPD) and matched healthy controls (HC). Data is shown as mean±SD. Refer to Table S6 for inferential statistics.

Exerkines and neurotrophic factors (Table S7-S13, Figure S5-S6).

Plasma clusterin. As seen in Table S7, plasma clusterin increased after MICT (p=0.005) and HIIT80% (p=0.012) for people with COPD, and HIIT60% in HC (p=0.009). While the Pre to Post change in clusterin tended to be lower in HIIT60% for COPD and MICT in HC, the change from Pre to MICT-Isotime or Post did not significantly differ between the sessions in any group (p=0.062 for COPD, p=0.224 for HC). Moreover, the change differed between the groups in HIIT60% where HC increased more than COPD (p=0.033) but not in the other sessions (p=0.807 for MICT-Iso, p=0.109 for MICT, p=0.611 for HIIT80%). The groups had similar levels of plasma clusterin with no between-group differences at any time point (p<0.05) except at post 30-minutes of HIIT60%, where those with COPD had lower values (Table S7).

Table S7. Plasma clusterin concentration ng/mL

		COPD		HC		P-Time	
		**N**	**Mean (SD)**	**N**	**Mean (SD)**	**COPD**	**HC**
**HIIT60%**	**Pre**	16	181724 (30538)	16	178589 (31712)	**<0.001**	**0.004**
	**Post**	15	187365 (31125)	16	194945 (34490)[a]		
	**30 min**	15	159778 (26135)[ab#]	16	179410 (26770)[b]		
**MICT**	**Pre**	16	165752 (42093)	16	175071 (25372)	**0.005**	**<0.001**
	**Iso**	12	182216 (37535)	16	185281 (30926)		
	**Post**	16	191428 (54947)[a]	16	178786 (33180)		
	**30 min**	16	172978 (39789)	16	162794 (26258)[bc]		
**HIIT80%**	**Pre**	16	170227 (36370)	15	176403 (27949)	**0.007**	0.057
	**Post**	16	191919 (41346)[a]	16	189664 (38094)		
	**30 min**	15	175298 (39618)	15	173542 (24146)		

Supramaximal high-intensity interval-training at 60% (HIIT60%) and 80% (HIIT80%) are performed at 60% and 80%, respectively of maximum mean power output for six seconds. Moderate intensity continuous training (MICT) is performed at 60% of maximal aerobic power. COPD: chronic obstructive pulmonary disease; HC: healthy control.
[a] = different from Pre (p<0.05), [b] = different from Post (p<0.05), [c] = different from Iso (p<0.05), # = different from HC (p<0.05)

In relative terms, the change from pre- to post-exercise in clusterin ranged from -7% to +22% (Table S8), depicted in Figure S5.

Table S8. Relative change in plasma clusterin concentration (ng/mL) from baseline.

		COPD		HC	
		**N**	**Mean (SD)**	**N**	**Mean (SD)**
**HIIT60%**	**Post**	15	1 (11)	16	10 (11)
	**30 min**	15	-14 (13)	16	1 (9)
**MICT**	**Iso**	12	17 (21)	16	6 (11)
	**Post**	16	18 (24)	16	2 (10)
	**30 min**	16	6 (16)	16	-7 (8)
**HIIT80%**	**Post**	16	22 (42)	16	-7 (8)
	**30 min**	15	11 (40)	15	0 (0)

Supramaximal high-intensity interval-training at 60% (HIIT60%) and 80% (HIIT80%) are performed at 60% and 80%, respectively of maximum mean power output for six seconds. Moderate intensity continuous training (MICT) is performed at 60% of maximal aerobic power. COPD: chronic obstructive pulmonary disease; HC:

healthy control

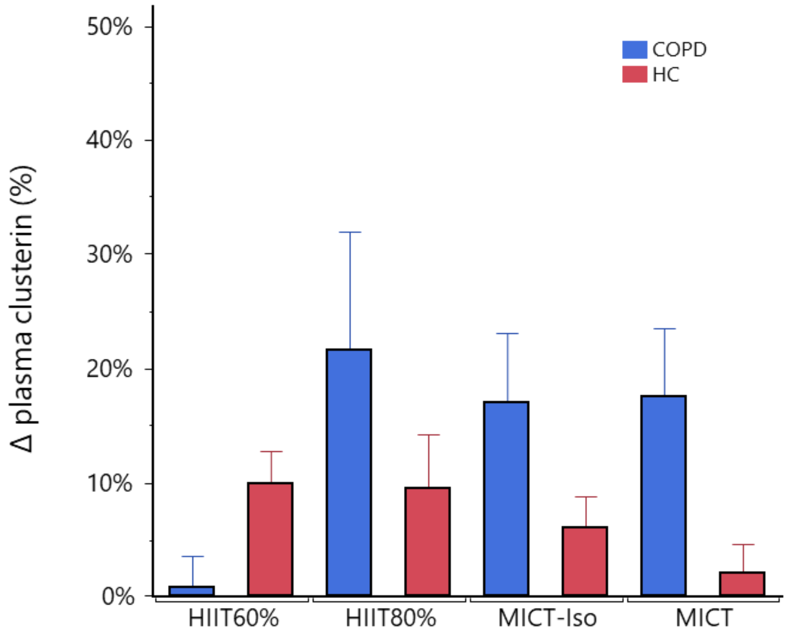

Figure S5. Relative change in plasma clusterin from baseline. Supramaximal high-intensity interval-training at 60% (HIIT60%) and 80% (HIIT80%) are performed at 60% and 80%, respectively of maximum mean power output for six seconds. Data is shown as mean±SE. Moderate intensity continuous training (MICT) is performed at 60% of maximal aerobic power. COPD: chronic obstructive pulmonary disease; HC: healthy control.

Serum BDNF. Serum BDNF increased in MICT and HIIT80% in HCs, but not in any session for those with COPD (Figure S6; Table S9). The change from Pre to Iso or Post did not differ between the sessions within any group (COPD: $p=0.977$; HC: $p=0.080$). Moreover, the change did not differ between the groups in any of the session (HIIT60%: $p=0.737$; MICT-Iso: $p=0.318$; MICT: $p=0.638$ for MICT; HIIT80%: $p=0.318$). The groups had similar levels of serum BDNF with no between-group differences at any time point ($p<0.05$).

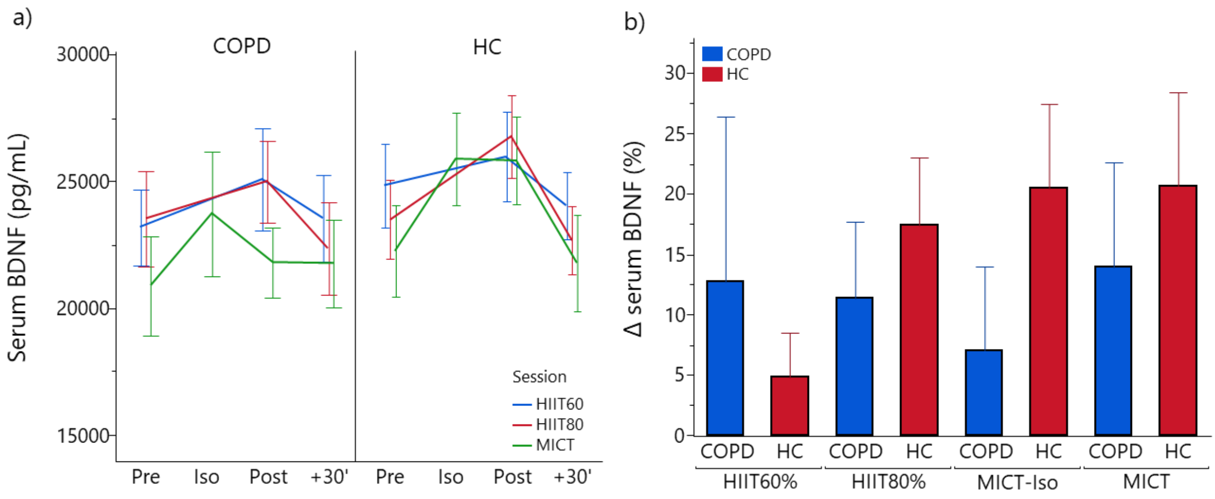

Figure S6. a) Exercise induced change in serum brain-derived neurotrophic factor during supramaximal high-intensity interval training (HIIT) and moderate-intensity continuous training (MICT) in people with chronic obstructive pulmonary disease (COPD) and matched healthy controls (HC). Data is shown as mean±SE. Panel **b)** shows the relative change (%) from baseline to post-exercise. See table S9 for statistical data. supramaximal HIIT at 60% of maximum mean power output for six-seconds; HIIT80: supramaximal HIIT at 80% of maximum mean power output for six-seconds.

Table S9. Serum brain-derived neurotrophic factor (sBDNF) concentration (pg/mL)

		COPD		HC		P-Time	
		N	Mean (SD)	N	Mean (SD)	COPD	HC
**HIIT60%**	**Pre**	16	23181 (6022)	16	24837 (6586)	0.729	0.106
	**Post**	15	25068 (7836)	16	25960 (7080)		
	**30 min**	15	23530 (6610)	16	24035 (5270)		
**MICT**	**Pre**	16	20880 (7805)	16	22245 (7255)	0.665	**<0.001**
	**Iso**	12	23716 (8475)	16	25877 (7253)[a]		
	**Post**	16	21795 (5462)	16	25815 (6895)[a]		
	**30 min**	16	21758 (6905)	16	21775 (7571)[bc]		
**HIIT80%**	**Pre**	16	23526 (7480)	16	23485 (6213)	0.071	**<0.001**
	**Post**	16	24981 (6407)	16	26755 (6492)[a]		
	**30 min**	16	22349 (7316)	16	22678 (5389)[b]		

Supramaximal high-intensity interval-training at 60% (HIIT60%) and 80% (HIIT80%) are performed at 60% and 80%, respectively of maximum mean power output for six seconds. Moderate intensity continuous training (MICT) is performed at 60% of maximal aerobic power. COPD: chronic obstructive pulmonary disease; HC: healthy control

[a] = different from Pre (p<0.05), [b] = different from Post (p<0.05), [c] = different from Iso (p<0.05)

Lactate. A modest increase (1.2-2.3 mM) in lactate was seen in both groups during all sessions (Figure 6; Table S10) in both groups. In COPD, the increase was higher during MICT than HIIT60%, but not HIIT80% (main effect of session: p=0.06; change in MICT vs. HIIT60%: p=0.09). For HCs, the increase in lactate was higher during both HIIT80% and MICT, compared to HIIT60% (main effect of session: p<0.001; change in HIIT80% and MICT vs. HIIT60%: p<0.001 for both). Yet, the groups had not statistically different levels of lactate at any time points (p<0.05).

Table S10. Lactate concentration (mM) during supramaximal high-intensity interval training and moderate-intensity continuous training.

		COPD		HC		P-Time	
		N	**Mean (SD)**	**N**	**Mean (SD)**	**COPD**	**HC**
**HIIT60%**	**Pre**	15	1.2 (0.4)	16	0.9 (0.3)	**<0.001**	**<0.001**
	**Post**	16	2.4 (0.6)[a]	16	2.2 (0.9)[a]		
	**30 min**	16	1.1 (0.3)[b]	16	1.0 (0.3)[b]		
**MICT**	**Pre**	16	1.0 (0.3)	16	1.0 (0.2)	**<0.001**	**<0.001**
	**Iso**	12	3.0 (0.7)[a]	16	3.2 (1.0)[a]		
	**Post**	16	2.9 (0.8)[a]	16	3.3 (1.3)[a]		
	**30 min**	16	1.2 (0.3)[bc]	16	1.3 (0.3)[bc]		
**HIIT80%**	**Pre**	16	1.0 (0.3)	16	1.0 (0.3)	**<0.001**	**<0.001**
	**Post**	16	2.8 (0.7)[a]	16	3.3 (1.2)[a]		
	**30 min**	16	1.2 (0.2)[b]	16	1.3 (0.3)[b]		

Supramaximal high-intensity interval-training at 60% (HIIT60%) and 80% (HIIT80%) are performed at 60% and 80%, respectively of maximum mean power output for six seconds. Moderate intensity continuous training (MICT) is performed at 60% of maximal aerobic power. COPD: chronic obstructive pulmonary disease; HC: healthy control

[a] = different from Pre (p<0.05), [b] = different from Post (p<0.05), [c] = different from Iso (p<0.05)

Plasma hepatocyte growth factor. While plasma HGF increased in all sessions prior to adjustment in change in plasma volume, the increase stayed significant for HIIT60% and MICT in the COPD group, and MICT for HCs (Figure 6, Table S11) after the adjustment. The change from Pre to Iso or Post did not differ between the sessions in any group (p=0.562 for COPD, p=0.161 for HC). Moreover, the change did not differ between the groups in any of the session (HIIT60%: p=0.836; MICT-Iso: p=0.445; MICT: p=0.450 for MICT; HIIT80%: p=0.210). The groups had similar levels of HGF at all time points (p<0.05, Table S11).

Table S11. Plasma hepatocyte growth factor (HGF) concentration (pg/mL)

		COPD		HC		P-Time	
		N	**Mean (SD)**	**N**	**Mean (SD)**	**COPD**	**HC**
**HIIT60%**	**Pre**	16	449 (842)	16	435 (1267)	**0.024**	0.078
	**Post**	15	798 (1570)[a]	15	469 (1343)		
	**30 min**	15	743 (1494)	15	478 (1425)		
**MICT**	**Pre**	16	497 (1144)	16	507 (1530)	**0.005**	**<0.001**
	**Iso**	12	972 (1720)[a]	16	581 (1779)[a]		
	**Post**	16	775 (1495)[a]	16	631 (1927)[a]		
	**30 min**	16	659 (1306)	16	470 (1367)		
**HIIT80%**	**Pre**	16	794 (1710)	16	397 (1114)	0.324	**0.012**
	**Post**	16	678 (1394)	16	607 (1885)		
	**30 min**	16	771 (1645)	15	393 (1096)[b]		

Supramaximal high-intensity interval-training at 60% (HIIT60%) and 80% (HIIT80%) are performed at 60% and 80%, respectively of maximum mean power output for six seconds. Moderate intensity continuous training (MICT) is performed at 60% of maximal aerobic power. COPD: chronic obstructive pulmonary disease; HC: healthy control
[a] = different from Pre (p<0.05), [b] = different from Post (p<0.05)

Plasma cathepsin B. Plasma levels of cathepsin B did not change during any session in any group (p<0.05, Table S12) and those with COPD had similar levels compared to HC with no differences at any time point.

Table S12. Plasma cathepsin B concentration pg/mL

		COPD		HC		P-Time	
		**N**	**Mean (SD)**	**N**	**Mean (SD)**	**COPD**	**HC**
**HIIT60%**	**Pre**	14	151 (58)	14	165 (86)	0.222	0.055
	**Post**	12	142 (58)	14	170 (87)		
	**30 min**	13	149 (60)	14	153 (81)		
**MICT**	**Pre**	14	158 (83)	14	207 (120)	0.394	0.103
	**Iso**	10	147 (85)	11	172 (102)		
	**Post**	14	157 (90)	14	181 (100)		
	**30 min**	14	158 (102)	13	177 (97)		
**HIIT80%**	**Pre**	13	163 (119)	13	172 (92)	0.689	0.536
	**Post**	14	153 (102)	14	153 (72)		
	**30 min**	14	166 (133)	12	167 (87)		

Supramaximal high-intensity interval-training at 60% (HIIT60%) and 80% (HIIT80%) are performed at 60% and 80%, respectively of maximum mean power output for six seconds. Moderate intensity continuous training (MICT) is performed at 60% of maximal aerobic power. COPD: chronic obstructive pulmonary

disease; HC: healthy control.

Serum irisin. Serum levels of irisin did not increase during any session in any group. Only a minor decrease after HIIT80% was seen in those with COPD (Table S13). Those with COPD had similar levels compared to HC, except for MICT-isotime where HCs had higher levels, which might be attributed to the change in sample size in the COPD group.

Table S13. Plasma irisin concentration pg/mL

		COPD		HC		P-Time	
		N	Mean (SD)	N	Mean (SD)	COPD	HC
**HIIT60%**	**Pre**	12	8.4 (2.0)	15	9.3 (2.4)	0.217	0.911
	**Post**	12	7.6 (2.8)	14	8.9 (2.5)		
	**30 min**	12	8.5 (2.7)	15	9.2 (2.7)		
**MICT**	**Pre**	12	7.5 (1.9)	15	9.0 (2.6)	0.240	0.701
	**Iso**	10	6.4 (1.8)[#]	15	9.0 (2.0)		
	**Post**	13	7.6 (2.7)	15	8.5 (1.9)		
	**30 min**	13	8.4 (2.6)	15	8.7 (2.6)		
**HIIT80%**	**Pre**	13	8.6 (2.3)	15	8.8 (2.4)	**0.033**	0.457
	**Post**	13	7.8 (2.1)	14	8.6 (2.3)		
	**30 min**	13	7.6 (2.6)[a]	15	9.5 (2.6)		

Supramaximal high-intensity interval-training at 60% (HIIT60%) and 80% (HIIT80%) are performed at 60% and 80%, respectively of maximum mean power output for six seconds. Moderate intensity continuous training (MICT) is performed at 60% of maximal aerobic power. COPD: chronic obstructive pulmonary disease; HC: healthy control. [a] = different from Pre (p<0.05), # = different from HC (p<0.05)

Inflammatory markers (Table S14-S19)

Plasma IL-6. Plasma IL-6 increased from baseline to 30-minutes post-exercise during all exercise in both groups (Figure 6, Table S14). For people with COPD, the change from pre-exercise to 30-minutes post exercise was greater in MICT than during HIIT60% (main effect of session: p=0.018; MICT vs HIIT60%: p=0.015) but not HIIT80% (p=0.186). For HCs, the increase was greater in MICT compared to both HIIT sessions (main effect of session: p<0.001; MICT vs. HIIT60%: p<0.001; MICT vs. HIIT80%: p=0.002). The change from baseline to post exercise in IL-6 did not differ between the groups in any of the session (p=0.505 for HIIT60%, p=0.201 for MICT-Iso, p=0.289 for MICT, p=0.092 for HIIT80%). However, participants with COPD tended to have higher levels of IL-6, reaching significance at some timepoints (Table S14).

Table S14. Plasma interleukin-6 concentration (pg/mL)

		COPD		HC		P-Time	
		N	Mean (SD)	N	Mean (SD)	COPD	HC
**HIIT60%**	**Pre**	15	1.50 (1.75)	14	0.88 (0.52)	**0.002**	**0.003**
	**Post**	14	1.63 (1.55)[#]	15	0.92 (0.75)		
	**30 min**	14	1.76 (1.42)[a]	15	1.22 (0.73)[ab]		
**MICT**	**Pre**	16	1.43 (1.36)[#]	16	0.81 (0.69)	**<0.001**	**<0.001**
	**Iso**	12	1.74 (1.65)[#]	16	1.07 (1.25)		
	**Post**	16	1.72 (1.60)[#]	16	1.11 (1.18)		
	**30 min**	16	2.14 (1.67)[abc]	16	1.99 (1.42)[abc]		
**HIIT80%**	**Pre**	16	1.38 (1.61)	14	0.75 (0.51)	**<0.001**	**0.002**
	**Post**	16	1.61 (1.50)[#]	15	0.81 (0.69)		
	**30 min**	16	1.77 (1.57)[ab]	15	1.18 (0.75)[ab]		

Supramaximal high-intensity interval-training at 60% (HIIT60%) and 80% (HIIT80%) are performed at 60% and 80%, respectively of maximum mean power output for six seconds. Moderate intensity continuous training (MICT) is performed at 60% of maximal aerobic power. COPD: chronic obstructive pulmonary disease; HC: healthy control
[a] = different from Pre (p<0.05), [b] = different from Post (p<0.05), [c] = different from Iso (p<0.05) # = different from HC (p<0.05)

Plasma IL-8. Plasma IL-8 increased significantly during HIIT60% in people with COPD but no other session (Table S15). The groups had similar levels of Interleukin-8 at all timepoints (p>0.05, Table S15).

Table S15. Plasma Interleukin-8 concentration (pg/mL)

		COPD		HC		P-Time	
		N	Mean (SD)	N	Mean (SD)	COPD	HC
**HIIT60%**	**Pre**	16	4.28 (2.17)	16	3.68 (1.21)	**0.006**	0.090
	**Post**	15	4.98 (2.86)[a]	16	3.92 (1.02)		
	**30 min**	15	4.54 (2.86)[b]	16	3.50 (0.97)		
**MICT**	**Pre**	16	4.73 (3.12)	16	3.89 (1.76)	0.512	0.400
	**Iso**	12	5.47 (2.58)	14	4.19 (1.54)		
	**Post**	16	4.83 (2.53)	16	4.61 (1.73)		
	**30 min**	16	4.65 (2.61)	16	4.22 (1.69)		
**HIIT80%**	**Pre**	16	4.76 (2.70)	16	3.96 (1.33)	0.450	0.174
	**Post**	16	4.79 (2.64)	16	4.35 (2.28)		
	**30 min**	15	4.39 (2.57)	16	3.68 (1.14)		

Supramaximal high-intensity interval-training at 60% (HIIT60%) and 80% (HIIT80%) are performed at 60% and 80%, respectively of maximum mean power output for six seconds. Moderate intensity continuous training (MICT) is performed at 60% of maximal aerobic power. COPD: chronic obstructive pulmonary disease; HC: healthy control
[a] = different from Pre (p<0.05), [b] = different from Post (p<0.05)

Plasma IL-10. Plasma IL-10 did not increase in any session for any group, only a small reduction was seen in COPD 30-minutes post HIIT80% ($p<0.05$, Table S16). The groups had similar levels of Interleukin-8 at all timepoints ($p>0.05$, Table S16).

Table S16. Plasma Interleukin-10 concentration (pg/mL)

		COPD		HC		P-Time	
		N	**Mean (SD)**	**N**	**Mean (SD)**	**COPD**	**HC**
**HIIT60%**	**Pre**	16	1.10 (2.27)	14	0.45 (0.16)	0.620	**0.037***
	**Post**	15	1.05 (2.21)	14	0.38 (0.14)		
	**30 min**	15	1.08 (2.34)	14	0.40 (0.16)		
**MICT**	**Pre**	16	1.18 (2.56)	16	0.41 (0.21)	0.185	0.516
	**Iso**	12	1.28 (2.92)	15	0.39 (0.17)		
	**Post**	16	1.11 (2.40)	14	0.40 (0.17)		
	**30 min**	16	1.14 (2.51)	16	0.39 (0.18)		
**HIIT80%**	**Pre**	16	1.16 (2.44)	15	0.41 (0.19)	**0.012**	0.136
	**Post**	16	1.16 (2.63)	15	0.37 (0.15)		
	**30 min**	16	1.09 (2.49)[a]	15	0.34 (0.17)		

Supramaximal high-intensity interval-training at 60% (HIIT60%) and 80% (HIIT80%) are performed at 60% and 80%, respectively of maximum mean power output for six seconds. Moderate intensity continuous training (MICT) is performed at 60% of maximal aerobic power. COPD: chronic obstructive pulmonary disease; HC: healthy control
*No significant difference between timepoints after adjustment for multiple comparisons.
[a] = different from Pre

Plasma IL-15. Plasma IL-15 levels were modest and did not significantly change during any session (Table S17). The groups had similar levels at all timepoints ($p<0.05$, Table S17).

Table S17. Plasma Interleukin-15 concentration (pg/mL)

		COPD		HC		P-Time	
		N	**Mean (SD)**	**N**	**Mean (SD)**	**COPD**	**HC**
**HIIT60%**	**Pre**	15	6.1 (5.7)	13	6.2 (4.6)	0.747	0.925
	**Post**	15	6.4 (5.4)	15	5.7 (3.6)		
	**30 min**	13	6.4 (4.8)	12	6.1 (4.3)		
**MICT**	**Pre**	13	6.5 (4.5)	14	6.6 (5.0)	0.572	0.525
	**Iso**	15	7.1 (5.7)	15	5.9 (3.0)		
	**Post**	13	6.5 (5.0)	15	6.2 (5.5)		
	**30 min**	16	6.5 (6.4)	12	6.1 (4.4)		
**HIIT80%**	**Pre**	14	6.1 (5.8)	14	5.3 (5.8)	0.441	0.069
	**Post**	15	6.0 (4.2)	14	9.4 (10.7)		
	**30 min**	16	7.0 (6.9)	13	5.7 (5.7)		

Supramaximal high-intensity interval-training at 60% (HIIT60%) and 80% (HIIT80%) are performed at 60% and 80%, respectively of maximum mean power output for six seconds. Moderate intensity continuous training (MICT) is performed at 60% of maximal aerobic power. COPD: chronic obstructive pulmonary disease; HC: healthy control

Plasma VEGF. Plasma VEGF did not increase during any session. Only a small decrease was change to post-30 minutes during HIIT80% in HC (Table S18). The groups had similar levels of VEGF-A at all timepoints (p<0.05, Table S18).

Table S18. Concentration of vascular-endothelial growth factor-a (VEGF-A) (pg/mL)

		COPD		HC		P-Time	
		**N**	**Mean (SD)**	**N**	**Mean (SD)**	**COPD**	**HC**
**HIIT60%**	**Pre**	16	16.8 (11.4)	16	17.0 (10.6)	0.097	0.9560
	**Post**	15	21.8 (18.0)	16	17.2 (12.5)		
	**30 min**	15	16.2 (11.4)	16	16.3 (10.5)		
**MICT**	**Pre**	16	16.8 (13.1)	16	16.9 (9.9)	0.076	0.2177
	**Iso**	11	25.7 (17.3)	16	19.2 (13.8)		
	**Post**	16	22.3 (19.3)	16	19.9 (14.9)		
	**30 min**	15	20.6 (13.0)	16	17.6 (12.4)		
**HIIT80%**	**Pre**	16	19.7 (14.4)	16	18.3 (11.7)	0.4506	**0.0153**
	**Post**	16	20.5 (17.4)	16	17.3 (11.7)		
	**30 min**	16	18.2 (12.9)	15	15.0 (10.3)[ab]		

Supramaximal high-intensity interval-training at 60% (HIIT60%) and 80% (HIIT80%) are performed at 60% and 80%, respectively of maximum mean power output for six seconds. Moderate intensity continuous training (MICT) is performed at 60% of maximal aerobic power. COPD: chronic obstructive pulmonary disease; HC: healthy control
[a] = different from Pre (p<0.05), [b] = different from Post (p<0.05)

Plasma TNF- α. There were no changes in TNF-α during any session for any of the groups (Table S19). The groups had similar levels of TNF-α at all timepoints (p<0.05).

Table S19. Concentration of tumour-necrosis factor-alpha (pg/mL)

		COPD		HC		P-Time	
		**N**	**Mean (SD)**	**N**	**Mean (SD)**	**COPD**	**HC**
**HIIT60%**	**Pre**	16	8.6 (2.7)	16	8.4 (3.5)	0.414	0.182
	**Post**	15	8.1 (2.3)	16	8.1 (3.2)		
	**30 min**	15	8.0 (2.2)	16	8.1 (3.1)		
**MICT**	**Pre**	16	8.5 (2.1)	16	8.1 (3.4)	0.915	0.768
	**Iso**	12	8.4 (1.6)	16	8.1 (3.0)		
	**Post**	16	8.3 (1.8)	16	8.0 (3.1)		
	**30 min**	16	8.2 (1.7)	16	8.2 (3.2)		
**HIIT80%**	**Pre**	16	8.5 (2.3)	16	8.3 (3.2)	0.298	0.601
	**Post**	16	8.3 (2.3)	16	8.2 (3.1)		
	**30 min**	16	8.2 (2.3)	15	8.2 (3.1)		

Supramaximal high-intensity interval-training at 60% (HIIT60%) and 80% (HIIT80%) are performed at 60% and 80%, respectively of maximum mean power output for six seconds. Moderate intensity continuous training (MICT) is performed at 60% of maximal aerobic power. COPD: chronic obstructive pulmonary disease; HC: healthy control

Supplementary references

1 GOLD. The Global Strategy for Diagnosis, Management and Prevention of COPD, 2024 report., (2024).

2 Miller, M. R. *et al.* Standardisation of spirometry. *Eur Respir J* **26**, 319-338 (2005). https://doi.org:10.1183/09031936.05.00034805

3 Wanger, J. *et al.* Standardisation of the measurement of lung volumes. *Eur Respir J* **26**, 511-522 (2005). https://doi.org:10.1183/09031936.05.00035005

4 Graham, B. L. *et al.* 2017 ERS/ATS standards for single-breath carbon monoxide uptake in the lung. *Eur. Respir. J.* **49** (2017). https://doi.org:10.1183/13993003.00016-2016

5 Hedenström, H., Malmberg, P. & Fridriksson, H. V. Reference values for lung function tests in men: regression equations with smoking variables. *Ups. J. Med. Sci.* **91**, 299-310 (1986). https://doi.org:10.3109/03009738609178670

6 Hedenström, H., Malmberg, P. & Agarwal, K. Reference values for lung function tests in females. Regression equations with smoking variables. *Bull. Eur. Physiopathol. Respir.* **21**, 551-557 (1985).

7 Quanjer, P. H. *et al.* Multi-ethnic reference values for spirometry for the 3-95-yr age range: the global lung function 2012 equations. *Eur. Respir. J.* **40**, 1324-1343 (2012). https://doi.org:10.1183/09031936.00080312

8 Koch, B. *et al.* Static lung volumes and airway resistance reference values in healthy adults. *Respirology* **18**, 170-178 (2013). https://doi.org:https://doi.org/10.1111/j.1440-1843.2012.02268.x

9 Michailopoulos, P., Kontakiotis, T., Spyratos, D., Argyropoulou-Pataka, P. & Sichletidis, L. Reference Equations for Static Lung Volumes and TL(CO) from a Population Sample in Northern Greece. *Respiration* (2015). https://doi.org:10.1159/000371469

10 Radtke, T. *et al.* ERS statement on standardisation of cardiopulmonary exercise testing in chronic lung diseases. *European Respiratory Review* **28**, 180101 (2019). https://doi.org:10.1183/16000617.0101-2018

11 Brudin, L., Jorfeldt, L. & Pahlm, O. Comparison of two commonly used reference materials for exercise bicycle tests with a Swedish clinical database of patients with normal outcome. *Clin. Physiol. Funct. Imaging* **34**, 297-307 (2014). https://doi.org:10.1111/cpf.12097

12 Gläser, S. *et al.* [The Study of Health in Pomerania (SHIP) reference values for cardiopulmonary exercise testing]. *Pneumologie* **67**, 58-63 (2013). https://doi.org:10.1055/s-0032-1325951

13 Gore, S., Blackwood, J., Guyette, M. & Alsalaheen, B. Validity and Reliability of Accelerometers in Patients With COPD: A SYSTEMATIC REVIEW. *J. Cardiopulm. Rehabil. Prev.* **38**, 147-158 (2018). https://doi.org:https://dx.doi.org/10.1097/HCR.0000000000000284

14 Spruit, M. A. *et al.* An official American Thoracic Society/European Respiratory Society statement: key concepts and advances in pulmonary rehabilitation. *Am. J. Respir. Crit. Care Med.* **188**, e13-64 (2013). https://doi.org:10.1164/rccm.201309-1634ST

15 Vogler, A. J., Rice, A. J. & Gore, C. J. Validity and reliability of the Cortex MetaMax3B portable metabolic system. *J. Sports Sci.* **28**, 733-742 (2010). https://doi.org:10.1080/02640410903582776

16 Robergs, R. A., Dwyer, D. & Astorino, T. Recommendations for improved data processing from expired gas analysis indirect calorimetry. *Sports Med.* **40**, 95-111 (2010). https://doi.org:10.2165/11319670-000000000-00000

17 Foster, C. *et al.* A new approach to monitoring exercise training. *J. Strength Cond. Res.* **15**, 109-115 (2001).

18 Dill, D. B. & Costill, D. L. Calculation of percentage changes in volumes of blood, plasma, and red cells in dehydration. *J. Appl. Physiol.* **37**, 247-248 (1974). https://doi.org:10.1152/jappl.1974.37.2.247

19 Hopkins, W. G., Marshall, S. W., Batterham, A. M. & Hanin, J. Progressive statistics for studies in sports medicine and exercise science. *Med. Sci. Sports Exerc.* **41**, 3-13 (2009). https://doi.org:10.1249/MSS.0b013e31818cb278
